# Reversible cold-induced lens opacity in a hibernator reveals a molecular target for treating cataracts

**DOI:** 10.1172/JCI169666

**Published:** 2024-09-17

**Authors:** Hao Yang, Xiyuan Ping, Jiayue Zhou, Hailaiti Ailifeire, Jing Wu, Francisco M. Nadal-Nicolás, Kiyoharu J. Miyagishima, Jing Bao, Yuxin Huang, Yilei Cui, Xin Xing, Shiqiang Wang, Ke Yao, Wei Li, Xingchao Shentu

**Affiliations:** 1 Eye Center, The Second Affiliated Hospital, School of Medicine, Zhejiang University, Hangzhou, China.; 2 Retinal Neurophysiology Section, National Eye Institute, National Institutes of Health, Bethesda, Maryland, USA.; 3 Department of Ophthalmology, Children’s Hospital, Zhejiang University School of Medicine, National Clinical Research Center for Child Health, Hangzhou, China.; 4 Department of Ophthalmology, Zhejiang Provincial People’s Hospital, People’s Hospital of Hangzhou Medical College, Hangzhou, China.; 5 College of Life Sciences, Peking University, Beijing, China.

**Keywords:** Ophthalmology, Stem cells, Ubiquitin-proteosome system

## Abstract

Maintaining protein homeostasis (proteostasis) requires precise control of protein folding and degradation. Failure to properly respond to stresses disrupts proteostasis, which is a hallmark of many diseases, including cataracts. Hibernators are natural cold-stress adaptors; however, little is known about how they keep a balanced proteome under conditions of drastic temperature shift. Intriguingly, we identified a reversible lens opacity phenotype in ground squirrels (GSs) associated with their hibernation-rewarming process. To understand this “cataract-reversing” phenomenon, we first established induced lens epithelial cells differentiated from GS-derived induced pluripotent stem cells, which helped us explore the molecular mechanism preventing the accumulation of protein aggregates in GS lenses. We discovered that the ubiquitin-proteasome system (UPS) played a vital role in minimizing the aggregation of the lens protein αA-crystallin (CRYAA) during rewarming. Such function was, for the first time to our knowledge, associated with an E3 ubiquitin ligase, RNF114, which appears to be one of the key mechanisms mediating the turnover and homeostasis of lens proteins. Leveraging this knowledge gained from hibernators, we engineered a deliverable RNF114 complex and successfully reduced lens opacity in rats with cold-induced cataracts and zebrafish with oxidative stress–related cataracts. These data provide new insights into the critical role of the UPS in maintaining proteostasis in cold and possibly other forms of stresses. The newly identified E3 ubiquitin ligase RNF114, related to CRYAA, offers a promising avenue for treating cataracts with protein aggregates.

## Abstract 2

RNF114 plays an key role in the reversal of hypothermic lens opacity in ground squirrels and may become a new therapeutic target for cataract

## Introduction

To perform normal biological functions, most proteins must exhibit appropriate folding and balanced turnover throughout their lifetime (1). The levels of different proteins in mammalian cells must be carefully controlled, and this state of a balanced proteome, referred to as protein homeostasis (or proteostasis) (2), depends on an extensive network of the ubiquitin-proteasome system (UPS), autophagy, and molecular chaperones and their regulators (3, 4). The organization of this network and its regulation in response to external and endogenous stresses are of fundamental importance in biology and medicine, as the failure to maintain proteostasis is associated with aging and numerous degenerative diseases (5, 6).

Recent interesting advances in stress adaptation have arisen from studies on hibernators, which have made it possible to understand the reversible protein quality control response to cold, a physical stress (7–9). Hibernators undergo a cyclical change of metabolic state involving a decrease in body temperature, from the awake state (~37°C) to the torpor state (~4°C) (8). As proteostasis is frequently disturbed in various pathological conditions (1), hibernators can be unique models for research on protein homeostasis. One hibernator, the thirteen-lined ground squirrel (GS; *Ictidomys tridecemlineatus*), has been extensively used because of the remarkable ability of its different organs and systems to withstand cold stress that causes non-hibernators to undergo severe organ failure (8, 10). For example, GS kidney tubular cells exhibit less cell death during sharp temperature changes than mouse kidney tubular cells (11), and the GS myocardium exhibits much greater apoptosis resistance than the mouse myocardium during hypothermia (12).

Strikingly, in the current study, lenses in GSs were found to have very different reactions from those of non-hibernators. In GSs, the lens became cloudy at 4°C but quickly turned transparent after rewarming to 37°C. However, in non-hibernators, including rats and mice, the lens became cloudy at 4°C and remained opaque for a long time after rewarming to 37°C (8 hours) (13). This phenomenon is relevant to cataracts, the most common cause of blindness worldwide, for which the only established treatment is surgical removal of the cloudy lens (14, 15). Disturbed proteostasis and abnormal protein aggregation caused by various factors are fundamental pathological changes in cataract formation (16, 17).

To explore the mechanism of the reversible temperature-dependent clarity change in GS lenses, we first constructed a GS derived–induced lens epithelial cell (iLEC) platform from GS-derived induced pluripotent stem cells (GS iPSCs). Here, we report a mechanism by which GS lenses reverse lens opacity via the UPS in the quality control of protein aggregates during rewarming. More importantly, we reveal that fast reversal of hypothermic cataracts in the rat lens can be reproduced by the RNF114 complex, a newly identified CRYAA-related E3 enzyme. The findings of our study reproduced the reversibility of cold-induced lens opacity in non-hibernators, thus demonstrating great promise for cataract clinical applications.

## Results

### GS lens opacity is reversed in the hypothermia-rewarming cycle.

**Figure 1:**
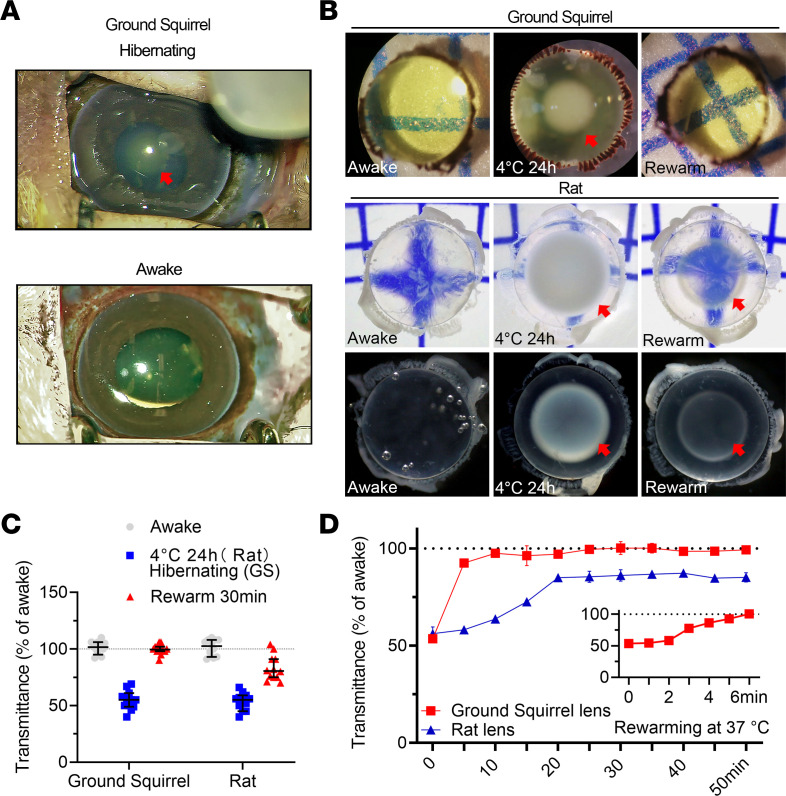
GS lens opacity is reversed during the hypothermia-rewarming cycle. (**A**) Photographs of the living squirrel (GS) lens in hibernating and awakened states. Red arrow indicates obvious lens opacity. (**B**) Images of dissected lenses under different conditions (untreated awake group, 4°C for 24 hours, and rewarmed at 37°C for 30 minutes; original magnification, ×4) captured using a Leica DM8000 microscope. GS: bottom light source; rat: bottom and side light sources. Red arrows indicate obvious lens opacities. (**C**) Quantitative results of the relative transmittance of ex vivo lenses in **B** (4 lenses per group, *n* = 3 independent experiments, mean ± SD, *P* < 0.01). (**D**) Statistical analysis of relative light transmittance in dissected lenses from rats and GSs rewarmed at 37°C for 0–50 minutes (recorded at 5-minute intervals, 4 lenses per group).

During hibernation/torpor, the GS lens became opaque; however, upon awakening, the lens quickly and completely recovered its transparency (Figure 1A). We observed this phenomenon in both 13-lined GSs and Mongolian ground squirrels. To confirm the unique adaptation of GSs, we compared the transparency of the isolated lenses of GSs and rats in response to hypothermia-rewarming treatment. In the physiological state, the rat lens was transparent and colorless; the GS lens was also transparent, but it was colored pale yellow. Both the GS and the rat lenses treated at 4°C for 24 hours developed obvious hypothermic cataracts with predominant central nuclear opacity. Subsequently, after about 5 minutes of rewarming process at 37°C, the opacities in GS lenses were completely eliminated, and the transmittance returned to the level before the low-temperature treatment. In contrast, the rat lens retained noticeable nuclear opacity, with transmittance restored to only 86%, and the prolonged rewarming time did not significantly improve the clarity further (Figure 1, B–D).

### Efficient differentiation of GS iPSCs into GS iLECs.

**Figure 2:**
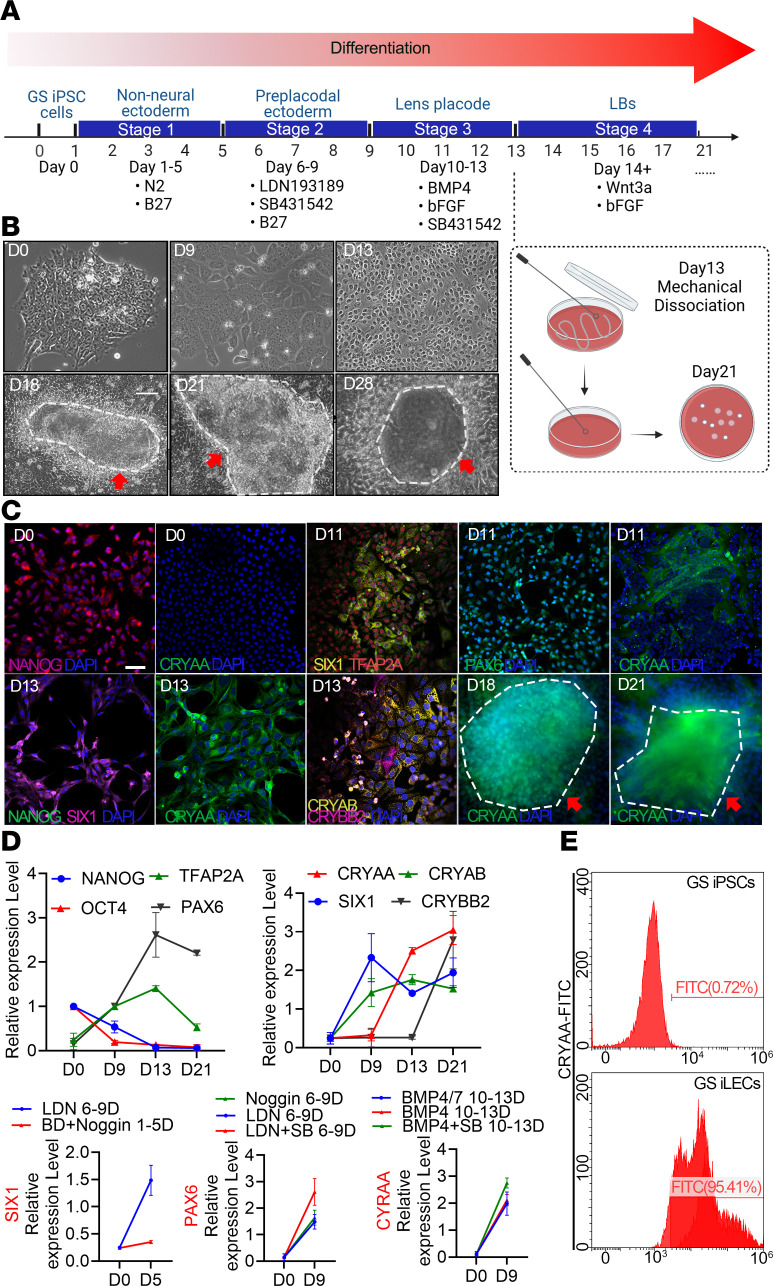
Efficient differentiation of GS iPSCs into GS iLECs. (**A**) Schematic representation of the 4-stage differentiation protocol from GS iPSCs to lens bodies (LBs). GS iLECs were obtained at the end of stage 3, and underwent 1 mechanical passaging during the transition from stage 3 to stage 4. (**B**) Cellular morphology at different stages of differentiation under a Leica optical microscope. Scale bar: 20 μm. Red arrows and dashed lines indicate LB-like structures during differentiation. (**C**) Immunofluorescence images showing the intracellular localization of fluorescently labeled characteristic proteins NANOG, CRYAA, SIX1, TFAP2A, PAX6, CRYAB, and CRYBB2 at different stages of differentiation. Scale bar: 60 μm. (**D**) qPCR analysis was conducted to assess the expression levels of stage-specific proteins and the impact of different differentiation protocols on the expression levels of each stage-specific protein (*n* = 3 independent experiments). (**E**) Proportion of CRYAA-positive cells detected by flow cytometry in GS iPSCs and GS iLECs (*n* = 3 independent experiments).

The lens is mainly composed of lens epithelial cells (LECs) and lens fiber cells. Only LECs have complete organelles and functions, which is crucial for lens metabolic stability and transparency (18, 19). To study GS lens adaptation strategies, we established a novel 4-stage protocol, as shown in Figure 2A, that was different from previous 3-stage human iPSC differentiation protocols (Yang et al., 2010, ref. 20; Li et al., 2016, ref. 21; Fu et al., 2017, ref. 22). This process involved transforming undifferentiated GS iPSCs into non-neural ectoderm, then to preplacodal ectoderm (PPE), to the lens placode (LP), and eventually to mature lens cells or tissue. We successfully obtained GS iLECs whose morphology resembled that of primary LECs (Figure 2B) with expression of the lens-specific marker αA-crystallin (CRYAA) (Figure 2C).

We aimed to increase the presence of SIX1^+^PAX6^+^ cells, since they represent the correct induction of PPE and have the potential to facilitate lens production (21). The previously established 3-step protocol induced the differentiation of neuroectoderm in GS iPSCs and led to the loss of PPE. Instead, we treated the cells early with medium containing N2/B27 followed by a combination of the small-molecule inhibitor LDN and SB431542 (a TGFB receptor inhibitor) to achieve non-neuroectoderm but PPE identities with higher levels of SIX1 and PAX6 at day 9. Next, to promote LP differentiation, we targeted transient BMP signaling as well as bFGF signaling (22, 23). By removing BMP7 from the 3-step protocol and using a combination of BMP4 and SB431542 (a promoter of epithelial cell conversion), we achieved efficient cell morphological conversion from day 10 to day 13 (Figure 2B), as corroborated by a high level of CRYAA expression (Figure 2, D and E). In our final stage, which differs from that of the 3-step protocol, we found that a single mechanical passaging of the cells can effectively generate colonies, create proper borders, facilitate the maturation of fibers, and eventually form lens bodies (LBs) (Figure 2B). This was confirmed by a significant downregulation of pluripotency markers and PPE markers (Figure 2D) and a further increase in the expression of crystallins CRYAA, CRYAB, and CRYBB2 (markers of more mature lens fibers), indicating maturation of lens differentiation at this stage (Figure 2, C and D). Overall, this new 4-step protocol yielded over 95% CRYAA-positive GS iLECs by day 13 (Figure 2E). It establishes an innovative GS iLEC platform derived from GS iPSCs, surpassing the production efficiency of previous differentiation protocols used for human iPSCs. This platform presents a robust in vitro model for in-depth mechanistic studies.

### The UPS promotes the degradation of aggregated crystallin.

**Figure 3:**
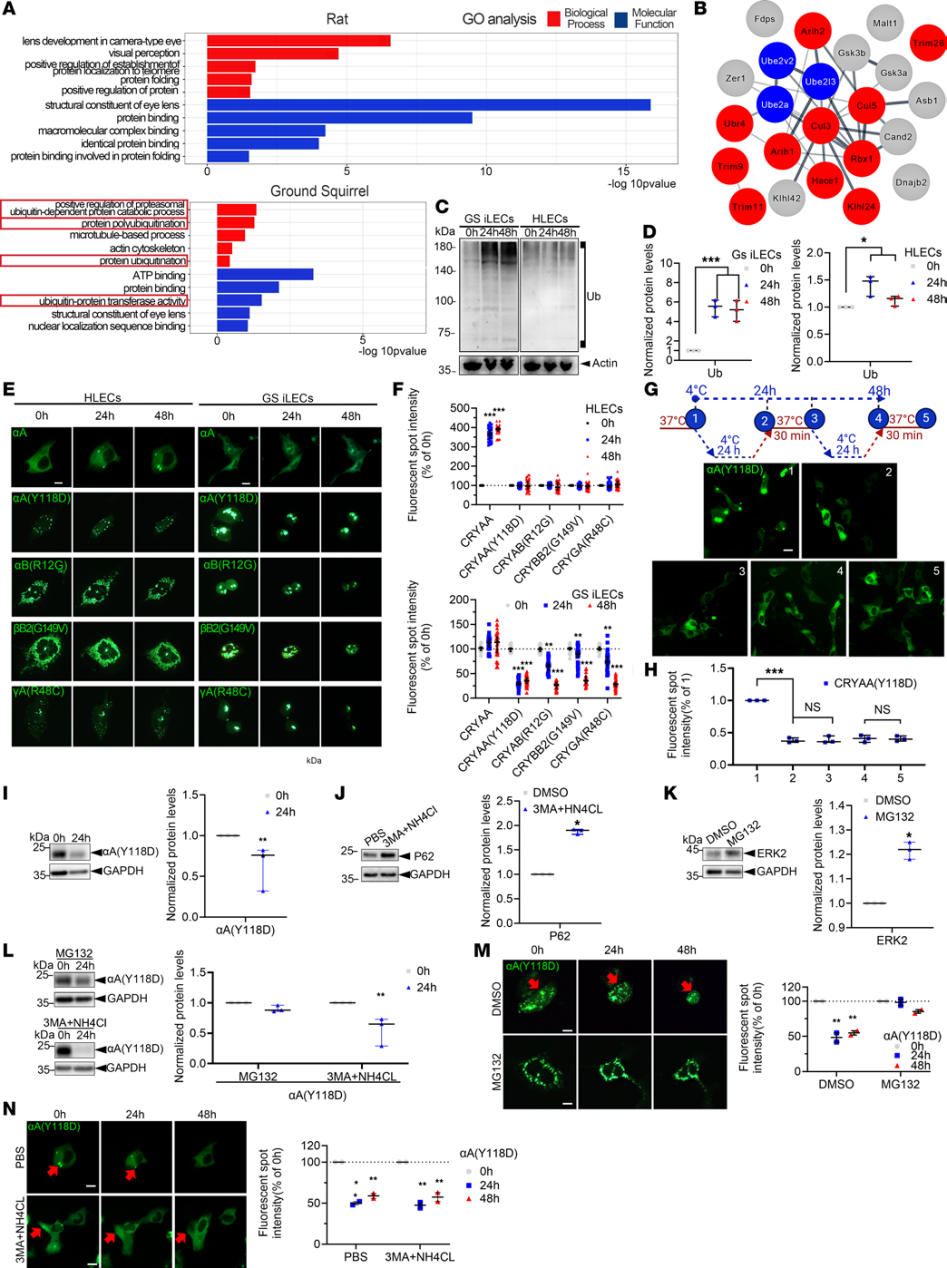
The UPS promotes the degradation of aggregated crystallin. (**A**) GO pathway analysis of upregulated proteins in GS and rat lens capsules after cold treatment. Red boxes: ubiquitin-related pathways. (**B**) Interactions among upregulated ubiquitin enzymes in GS lens capsules post-cold treatment. Gray: E1, blue: E2, red: E3. (**C**) Immunoblotting to detect expression levels of total ubiquitin protein in GS iLECs and HLECs after 30 minutes of rewarming following 24 and 48 hours of cold treatment. (**D**) Corresponding statistical graph of **C** (1-way ANOVA, *n* = 3 independent experiments). (**E**) Localization of GFP-tagged mutant crystalline proteins in HLECs and GS iLECs after cold treatment and rewarming. Scale bars: 8 μm. (**F**) Statistical analysis of fluorescence spot intensity in the live-cell imaging experiment described in **E** (2-tailed Student’s *t* tests followed by Holm-Šidák correction, *n* = 3, 10 cells per experiment). (**G** and **H**) Live-cell imaging of GS iLECs showing intracellular localization and relative fluorescence intensity of exogenous αA (Y118D) under conditions 1–5 (1-way ANOVA, *n* = 3, 5 fields of view per experiment). Scale bar: 20 μm. (**I**–**L**) Immunoblotting to detect expression levels of relevant proteins (2-tailed Student’s *t* test, *n* = 3 independent experiments). (**I**) Protein levels of exogenous CRYAA(Y118D) in GS iLECs after 24 hours of low-temperature rewarming. (**J**) Effect of 3MA+NH_4_Cl on p62 protein levels. PBS treatment used as control. (**K**) Effect of MG132 on ERK2 protein levels; DMSO treatment used as control. (**L**) Protein levels of exogenous CRYAA(Y118D) in GS iLECs after 24 hours of low-temperature rewarming. (**M** and **N**) CRYAA (Y118D) localization in GS iLECs after cold treatment and rewarming. Scale bars: 8 μm. Red arrows indicate prominent protein aggregates. Intensity of intracellular fluorescence spots was also quantified (2-tailed Student’s *t* tests followed by Holm-Šidák correction, *n* = 3, 10 cells per experiment). (All values are represented as mean ± SD, with *P < 0.05, **P < 0.01, and ***P < 0.001 indicating statistical significance.)

**Figure 4:**
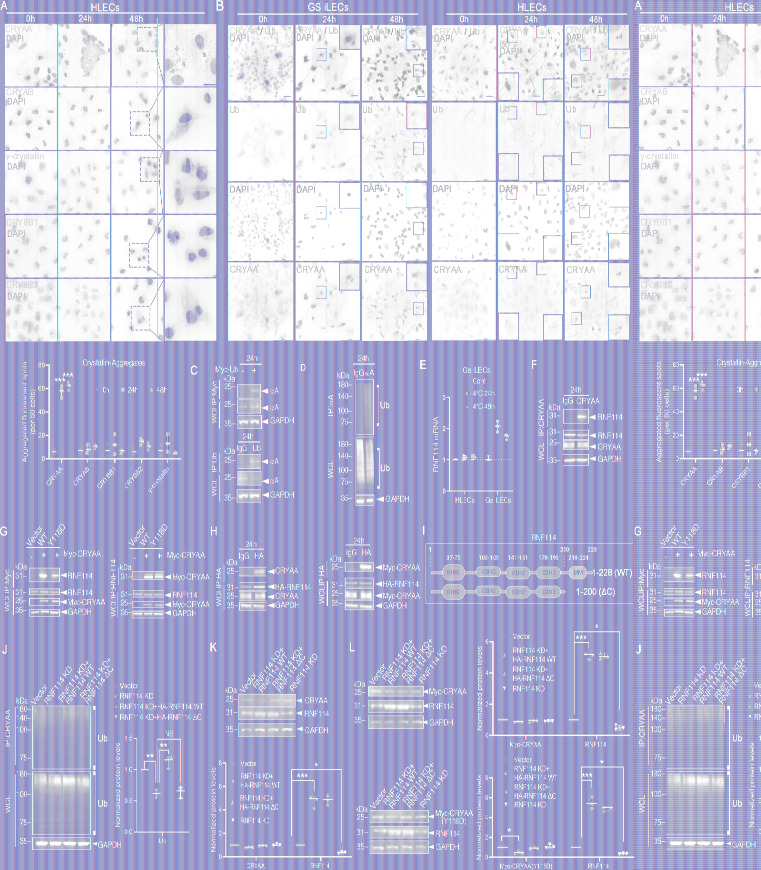
RNF114 promotes CRYAA ubiquitination during hypothermia-rewarming in GS iLECs. (**A**) Intracellular localization of α-, β-, and γ-crystallins in HLECs after cold treatment and rewarming. Red arrows: protein aggregates. Scale bars: 20 μm. Intensity of intracellular fluorescence spots was also quantified (2-tailed Student’s *t* test, *n* = 3, 50 cells per experiment). (**B**) Colocalization of CRYAA and Ub in GS iLECs and HLECs after cold treatment. Scale bars: 40 μm. (**C** and **D**) Immunoprecipitation assay to detect interaction between CRYAA and ubiquitin. Exogenous Myc-ubiquitin with αA. Endogenous ubiquitin with αA. Controls with goat anti-mouse IgG–coated magnetic beads are included. (*n* ≥ 3 independent experiments.) (**E**) qPCR analysis to assess the level of RNF114 mRNA in HLECs and GS iLECs after low-temperature treatment (*n* = 3 independent experiments). (**F**–**H**) CRYAA-RNF114 interaction in GS iLECs after cold rewarming. (**F**) Endogenous CRYAA with RNF114. (**G**) Exogenous CRYAA with RNF114. “Vector” refers to the transfection of the empty vector pCEP4-tetR. (**H**) Exogenous RNF114 with CRYAA. (**I**) Construction of the C-terminal truncated recombinant protein RNF114ΔC (1–200 aa). UIM, ubiquitin interaction motif. (**J**) Immunoprecipitation assay to investigate the interaction between endogenous CRYAA and ubiquitin, as well as their regulation by RNF114. “RNF114 KD” refers to the transfection of RNF114 siRNA to knock down the expression of RNF114. “RNF114 KD + RNF114 WT/ΔC” refers to the transfection of RNF114 WT/ΔC plasmid into cells where RNF114 has been knocked down. (2-tailed Student’s *t* tests followed by Holm-Šidák correction, *n* = 3 independent experiments.) (**K** and **L**) Immunoblotting was used to assess expression levels of endogenous CRYAA (**K**), exogenous CRYAA WT, and the Y118D mutant (**L**) in GS iLECs under normal temperature conditions, subject to RNF114 regulation (2-tailed Student’s *t* tests followed by Holm-Šidák correction, *n* = 3 independent experiments). (All values are presented as mean ± SD, *P < 0.05, **P < 0.01, ***P < 0.001.)

To further explore the specific mechanisms of cold adaptation in the lenses of hibernating animals, we used TMT–liquid chromatography–tandem mass spectrometry–based proteomics to examine the lens capsule tissues containing LECs from hibernating GSs and non-hibernating rats. Differential expression analysis was performed using the R package DESeq2, followed by Gene Ontology (GO) enrichment analysis and visualization with R to identify proteomic differences between the lens tissues of hibernators and non-hibernators. Interestingly, ubiquitin-related GO terms, including protein ubiquitination, ubiquitin-protein ligase activity, and proteasomal ubiquitin-dependent protein catabolic process, were significantly enriched only in GSs after rewarming (Figure 3A). Various E1, E2, and E3 enzymes involved in the UPS process were markedly upregulated (Figure 3B). In contrast, in rats, there was a notable enrichment in GO terms related to molecular chaperone–mediated protein folding. According to these results, we suspected that the mechanism of opacity reversal in the GS lens during the hypothermia-rewarming cycle might be related to protein degradation mediated by the ubiquitin-proteasome pathway. Indeed, we confirmed that the hypothermia-rewarming treatment significantly elevated the ubiquitination level in GS iLECs compared with human LECs (HLECs) (Figure 3, C and D). It is noteworthy that the formation of aggregated proteins and their ubiquitin-mediated degradation are a dynamic process. This may explain why the degradation of these extensively accumulated ubiquitinated proteins in GS iLECs was still ongoing at the 30-minute time point we examined after rewarming (Figure 4B, where the fluorescence intensity of labeled ubiquitin remains significantly higher than that in the control group after 30 minutes of rewarming following 24 and 48 hours of cold treatment). However, we believe that further extending the rewarming period might allow the ubiquitination levels to return to normal. Additionally, we introduced GFP-tagged CRYAA WT into both HLECs and GS iLECs, and observed no formation of aggregates within either cell type. After 24 and 48 hours of hypothermia treatment and rewarming for 30 minutes, distinct aggregates were only observed in HLECs but not GS iLECs. In contrast, when we performed knockin of GFP-tagged mutant crystallins in HLECs and GS iLECs, including the identified cataract-associated mutants CRYAA(Y118D), CRYAB(R120G), CRYBB2(G149V), and CRYGA(R48C) (23–26), we observed the aggregated fluorescent spots representing the mutant crystallins in both cells (Figure 3, E and F). After hypothermia-rewarming treatment, the number of fluorescent spots as well as the fluorescence intensity aggregated for all types of mutant crystallins was dramatically decreased in GS iLECs, but not in HLECs. Further investigation into the hypothermia-rewarming process revealed a significant reduction in the intensity of aggregates for CRYAA(Y118D) in GS iLECs after 24 hours of cold treatment. This reduction remained consistent during subsequent rewarming treatment processes and repeated cycles of hypothermia-rewarming, indicating that the primary effect of the cold treatment process was the elimination of aggregates in GS iLECs (Figure 3, G and H).

It is widely accepted that proteins are degraded through the UPS or autophagy in mammalian cells (9). Using inhibitors of these two pathways, we observed that reduction in levels of the mutant CRYAA(Y118D) after rewarming was markedly inhibited by the proteasome inhibitor MG132 but not by 3MA or NH_4_Cl (PI3K inhibitor/lysosomal inhibitor; Figure 3, I–L). Further, live-cell imaging also showed that MG132, rather than 3MA or NH_4_Cl, inhibited the elimination of mutant αA-crystallin (Figure 3, M and N) and αB-crystallin (Supplemental Figure 1; supplemental material available online with this article; https://doi.org/10.1172/JCI169666DS1) fluorescent spots in GS iLECs. These observations indicate that the UPS in proteasome but not autophagy in lysosome is critical for the clearance of mutant αA-crystallin.

### RNF114 promotes CRYAA ubiquitination during hypothermia-rewarming in GS iLECs.

Our results on the stability of endogenous crystallins showed that only CRYAA formed significant aggregates during the cryo-rewarming cycle; CRYAB, CRYBB1, CRYBB2, CRYBB3, and γ-crystallins did not aggregate significantly (Figure 4A). Therefore, for endogenous CRYAA, we subsequently confirmed its interaction with ubiquitin through immunofluorescence and coimmunoprecipitation (co-IP) (Figure 4, B–D). Following rewarming, an increase in polyubiquitination levels of CRYAA in GS iLECs was observed via co-IP. In the CRYAA pull-down assays, ubiquitination bands appeared smeared because of variations in polyubiquitination sites or chain lengths during the ubiquitination process, resulting in a trailing effect during gel electrophoresis separation. Ubiquitin colocalization with CRYAA fluorescent spots was further verified by immunofluorescence. The specific colocalization of CRYAA with ubiquitin in GS iLECs, as opposed to HLECs, serves as indirect evidence to exclude potential biases in co-IP results due to CRYAA’s binding to ubiquitinated substrates, attributable to its chaperone function. Ubiquitination, defined as a series of enzymatic cascades, is promoted by 3 different enzyme types, among which E3 ubiquitin ligases determine the substrate specificity and facilitate the transfer of ubiquitin onto targets (27). To identify potential E3 ubiquitin ligases that interact with CRYAA during hypothermia-rewarming, we used endogenous CRYAA pull-down and mass spectrometry experiments. We identified more than 100 proteins that specifically bind to CRYAA in the rewarmed group, one of which, called RNF114, has been reported to have E3 ubiquitin ligase activity (Supplemental Table 4). The following experiments were conducted to further investigate the impact of RNF114 on the reversal of lens opacity. First, the mRNA levels of RNF114 were elevated only in GS iLECs, not in HLECs, after hypothermia-rewarming treatment, indicating that RNF114 might play a specific role in the adaptive regulation of GS lenses (Figure 4E). Co-IP further confirmed the interaction between RNF114 and CRYAA in GS iLECs after rewarming (Figure 4, F–H). Knockdown of RNF114 resulted in reduced polyubiquitination of CRYAA, while the overexpression of RNF114 WT significantly facilitated the reduction in levels of CRYAA(Y118D). It is noteworthy that we constructed RNF114ΔC (1–200), which lacks the ubiquitin interaction motif domain essential for ubiquitin binding (28) (Figure 4I). The introduction of RNF114ΔC failed to enhance the turnover of CRYAA (Figure 4, I–L).

### RNF114 promotes CRYAA proteasomal degradation in HLECs.

**Figure 5:**
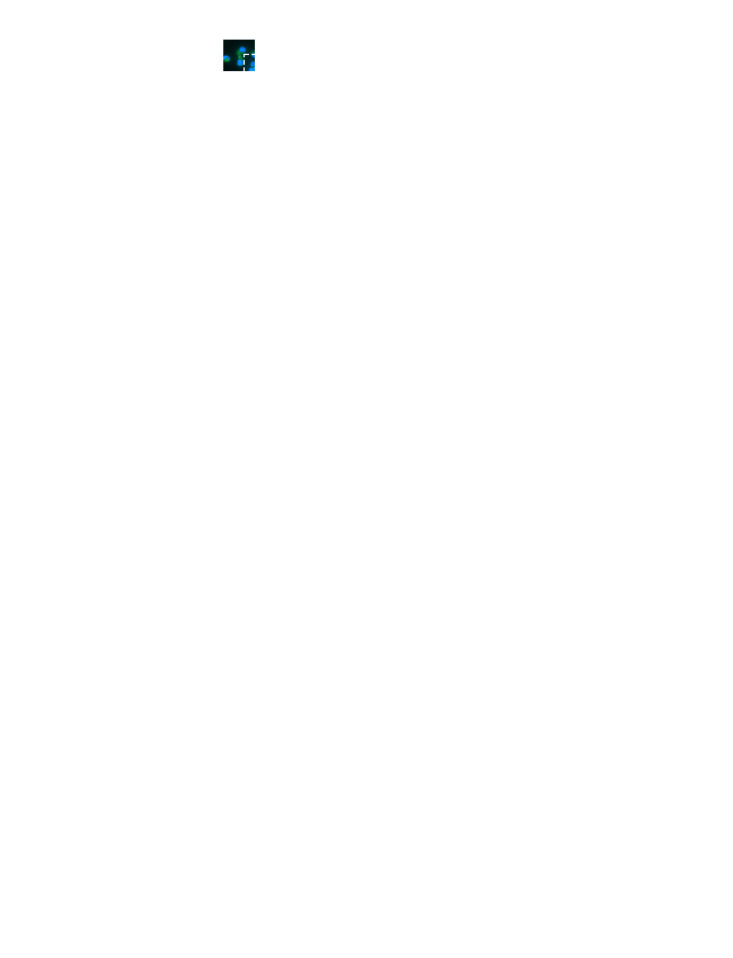
RNF114 promotes CRYAA proteasomal degradation in HLECs. (**A**) Immunofluorescence images depict the intracellular localization of endogenous CRYAA in GS iLECs, HLECs, and HLECs transfected with RNF114 WT or RNF114 ΔC after 24 or 48 hours of low-temperature treatment followed by 30 minutes of rewarming. Red arrows indicate prominent protein aggregates. Scale bars: 20 μm. (**B**) Statistical analysis of fluorescence spot intensity from **A** (2-tailed Student’s *t* test, *n* = 3 independent experiments, 50 cells per experiment). (**C**) Immunoblotting to detect protein levels of endogenous CRYAA in cells subjected to the aforementioned treatments. (**D**) Statistical analysis of protein expression levels from **C** (2-tailed Student’s *t* tests followed by Holm-Šidák correction, *n* = 3 independent experiments). (**E**) qPCR analysis to measure mRNA expression levels of CRYAA in GS iLECs after 24 hours of low-temperature rewarming and subsequent statistical analysis (*n* = 3 independent experiments). (**F**) Live-cell imaging was used to assess the impact of ubiquitin system inhibition on the function of exogenous RNF114 WT in relation to WT CRYAA and Y118D. The effects of overexpressing RNF114 WT and RNF114 ΔC on the intracellular localization of Y118D in HLECs were also observed. Scale bars: 8 μm. (**G**) Statistical analysis of fluorescence spot intensity from **F** (*n* = 3 independent experiments, 10 cells per experiment). (**H**) Immunoblotting to detect expression levels of knocked-in CRYAA(Y118D) in treated HLECs to validate the RNF114-mediated reduction mechanism via ubiquitin (2-tailed Student’s *t* tests followed by Holm-Šidák correction, *n* = 3 independent experiments). (All values are presented as mean ± SD, *P < 0.05, ***P < 0.001.)

According to the differences in the reversibility of lens opacity between hibernating animals and non-hibernating animals, including humans, we explored the expression of endogenous CRYAA in HLECs and GS iLECs after hypothermia-rewarming treatment. After rewarming, the fluorescent spot intensity representing aggregated CRYAA was higher in HLECs than in GS iLECs, and the duration of cold exposure correlated with increased levels of observed CRYAA aggregation. Knockin of RNF114 WT, but not RNF114ΔC, significantly eliminated these aggregates (Figure 5, A and B). Given previous research establishing the relationship between RNF114 and CRYAA ubiquitination in GS iLECs, we sought to determine whether RNF114 could also influence the turnover of CRYAA in HLECs. Immunoblotting results indicated a substantial decrease in endogenous CRYAA levels in GS iLECs and HLECs overexpressing RNF114 WT after rewarming, whereas no significant decrease was observed in untreated or RNF114ΔC-overexpressing HLECs (Figure 5, C–E). Live-cell imaging and immunoblotting further confirmed this effect in HLECs with CRYAA(Y118D) knockin, which was attenuated by MG132 treatment (Figure 5, F–H).

### RNF114 reverses cataracts in non-hibernators.

**Figure 6:**
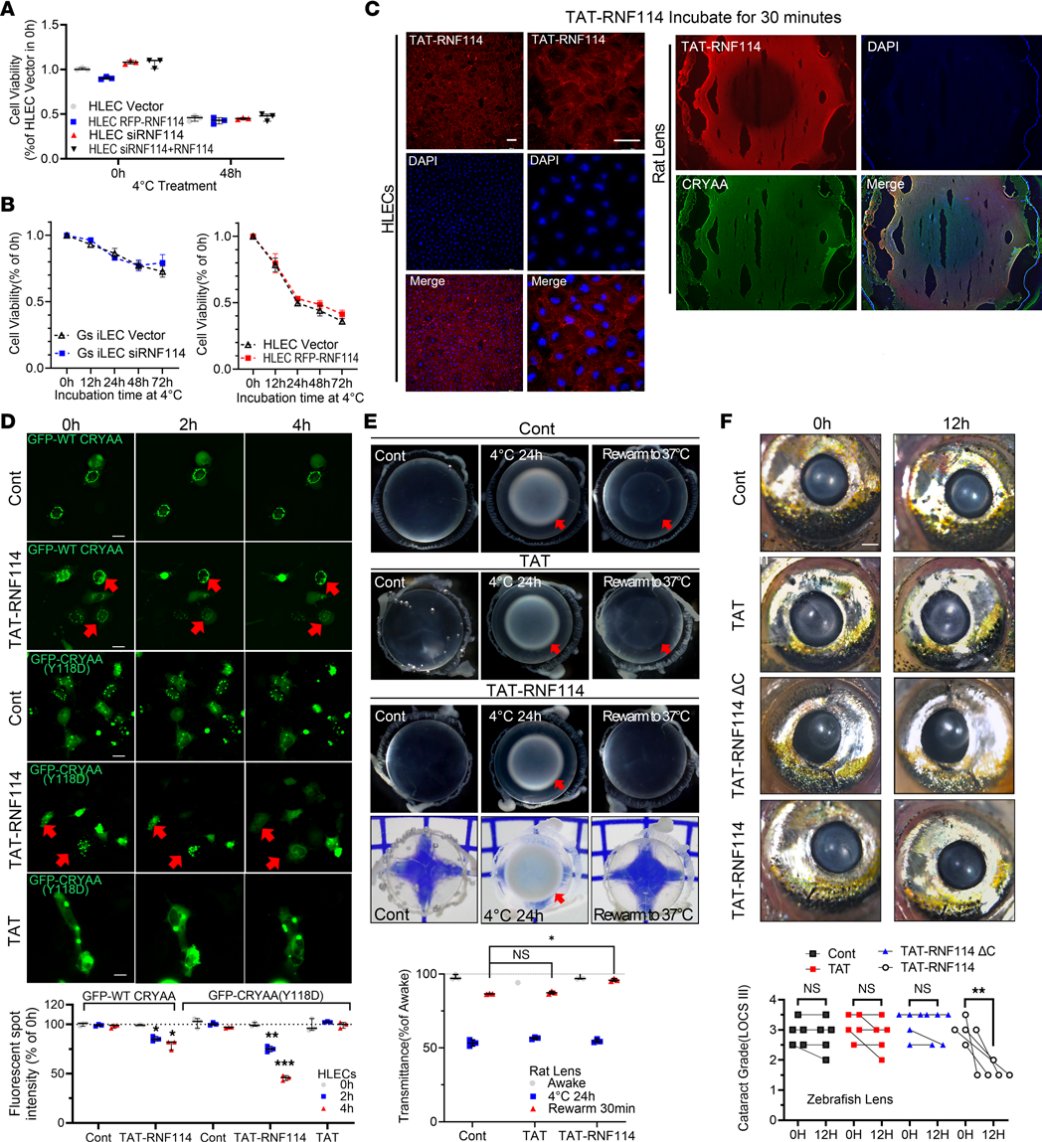
RNF114 reverses cataracts in non-hibernators. (**A** and **B**) CCK8 assay was performed to evaluate cell viability of RNF114-knockin or -knockdown HLECs and GS iLECs under low-temperature treatment, followed by statistical analysis (*n* = 3 independent experiments). (**C**) Immunofluorescence images depict localization of TAT-RNF114 in HLECs and rat lenses after 30 minutes of TAT-RNF114 pretreatment. Scale bars: 20 μm. Rat lens slices: original magnification, ×4. (**D**) Live-cell imaging showing intracellular localization of GFP-CRYAA(Y118D) in HLECs after 30 minutes of pretreatment. GFP–WT CRYAA: treated with TAT-RNF114 (100 μM); GFP-CRYAA(Y118D): treated with TAT-RNF114 (100 μM) or TAT (100 μM). Red arrows indicate typical protein aggregate degradation. Intensity of intracellular fluorescence spots was also quantified (2-tailed Student’s *t* tests followed by Holm-Šidák correction, *n* = 3, 5 fields of view per experiment). (**E**) Photographs of dissected rat lenses pretreated with TAT (100 μM) or TAT-RNF114 (100 μM) (awake group, 4°C for 24 hours, and rewarmed at 37°C for 30 minutes). For TAT and TAT-RNF114 treatments: TAT or TAT-RNF114 was added to the culture medium containing dissected rat lenses in the last 30 minutes of low-temperature treatment. Control and TAT groups: bottom light source imaging; TAT-RNF114: both bottom and side light source imaging. Red arrows indicate noticeable lens opacities. Original magnification, ×4. Quantitative analysis of relative light transmittance in dissected lenses (2-tailed Student’s *t* tests followed by Holm-Šidák correction, 4 lenses per group). (**F**) TAT (100 μM), TAT-RNF114ΔC (100 μM), or TAT-RNF114 (100 μM) was added to the live zebrafish culture dish induced with H_2_O_2_ to observe lenses after 0 and 12 hours of drug treatment under a Leica DM8000 microscope. Scale bar: 1.0 mm. Original magnification, ×2. Qualitative scoring of zebrafish lens transparency improvement based on LOCS III grading system through visual analysis, and statistical representation showing changes in lens transparency for each zebrafish (2-tailed Student’s *t* test, 1 eye from 5 zebrafish per group). (All values are presented as mean ± SD, *P < 0.05, **P < 0.01, ***P < 0.001.)

Having shown the potential of RNF114 in CRYAA turnover during hibernation adaptation, we examined the cytotoxicity of RNF114. We found that RNF114 did not affect the cell viability of either HLECs or GS iLECs (Figure 6, A and B), validating the safety of using RNF114 for the treatment of hypothermic cataracts. To facilitate the delivery of RNF114 to HLECs and rat lenses, we conjugated RNF114 with TAT-(47–58), a cell-penetrating peptide derived from the HIV-1 regulatory protein TAT that has been validated for use in multiple systems, including the eye (29–31). Immunofluorescence images confirmed the entry of TAT-RNF114 into the cytoplasm of HLECs and isolated rat lenses after a 30-minute incubation (Figure 6C). Next, we incubated TAT or TAT-RNF114 with GFP-CRYAA(Y118D) knockin HLECs for 4 hours and observed a gradual reduction in aggregated fluorescent spots in the cytoplasm, particularly in CRYAA(Y118D) HLECs (Figure 6D). We then developed the rat hypothermic cataract model by incubating rat lenses at 4°C for 24 hours and observed rapid opacity reversal in the TAT-RNF114–treated lenses, while the nuclei of the control lenses remained opacified. Lens transmittance measurement also verified that treatment with TAT-RNF114, but not TAT, significantly improved the rat lens transmittance efficiency during hypothermia-rewarming (Figure 6E). In addition, we induced cataracts in zebrafish with H_2_O_2_ — a form of oxidated stress instead of cold stress. We observed a reduction in lens opacity after 12 hours of TAT-RNF114 treatment, whereas the TAT-RNF114ΔC and TAT control groups did not show this effect, and 4 of 5 zebrafish had at least 1 grade of transparency improvement on the Lens Opacities Classification System III (LOCS III) (Figure 6F), indicating that RNF114 can alleviate lens opacity induced by stresses other than cold.

## Discussion

Misfolded or aggregated proteins must be degraded to avoid cellular damage, and extensive degradation networks cooperate to maintain proteostasis and adapt to environmental changes (1, 32, 33). However, the underlying networks in different conditions remain to be clarified. Here we demonstrate a mechanism by which GSs reverse lens opacity that relies mainly on UPS upregulation of CRYAA aggregate during rewarming. A newfound CRYAA E3 ubiquitin ligase, RNF114, plays a central role in aggregate degradation and turnover and proves to have significant reversible effect on different stress-induced cataract models.

Mechanistic studies on remarkable hibernation-related features in animals remain a challenge. We took advantage of the recently developed GS iPSCs and successfully differentiated them into GS iLECs. Unlike the 3-stage protocols for differentiation of human iPSCs into lens tissue described previously (20–22), our protocol uses a hybrid of small molecules and transcription factors to efficiently and reproducibly differentiate GS iPSCs into GS iLECs, creating a lens research platform for exploration of hypothermic cataracts. Consistent with the reports of Reichert et al. (34) and Leung et al. (35), we found that the derivation of GS LPs requires first a transition from iPSCs to non-neuroectoderm and subsequently acquisition of preplacodal ectoderm (PPE) and APE identities. By shortening Noggin treatment and adding a small molecule, SB431542, we successfully suppressed neuroectoderm formation and promoted epithelial phenotype. In addition to GS iLECs, other cell types, such as lens fiber cells derived at the final stage, PPE lineage–derived olfactory epithelial cells, anterior pituitary cells, paired cranial ganglia neurons, and inner ear epithelial cells derived through modulation of BMP signaling, can also be obtained. Thus, this platform provides ample research opportunities for evaluating cold stress adaptation in a wider range of tissues.

The proteostasis network, which integrates general and specialized chaperone components to ensure proper protein folding and trafficking for disaggregation and proteolytic degradation of misfolded proteins, relies mainly on the UPS and the autophagy system (32, 36, 37). Our data demonstrated that the autophagy inhibitors 3MA and NH_4_Cl did not change the reduction levels of cataract-associated crystallin mutant aggregates in GS iLECs, while the proteasome inhibitor MG132 greatly decreased the degradation level. Interestingly, the GO terminology analysis of proteomics and cell blots also implied that the UPS system may be the key process for maintaining proteostasis in hibernators. In contrast, rats may primarily rely on chaperone activity to maintain protein folding. High concentrations of crystallin proteins in the lens contribute to lens transparency and refractive properties (38). The crystallin superfamily is divided into 2 major families, α and βγ (39). Both of them are reported to be associated with cataracts (40). However, we found that αA-crystallin is the only endogenous crystallin that forms aggregates in the hypothermia-rewarming cycle. The α-crystallins are composed of 2 gene products, CRYAA and CRYAB, which are also members of the small heat shock protein (HSP20) family and have molecular chaperone functions that promote proteostasis. CRYAA is preferentially restricted to the lens, while CRYAB is expressed widely in many tissues and organs, such as the heart and muscle (41, 42). Compared with CRYAB, CRYAA is more prone to aggregation and exhibits significantly lower chaperone activity at low temperatures (43). This implies that the rat crystalline lens, which overly relies on this mechanism for protein homeostasis, is hindered in maintaining lens transparency. In contrast, in GS lenses, CRYAA aggregates might be the main substrate for the UPS in the hypothermia-rewarming cycle, while other crystallins have more stable cold adaptation and conserved proteostasis functions. Further exploration of the mechanisms in other organs underlying the degradation of the crystallin protein family is warranted.

In the UPS process, E3 ubiquitin ligases interact with substrates with high specificity and control their ubiquitination (27). In the lens, the UPS selectively recognizes and degrades crystallin proteins with abnormal structures, including oxidized, glutathiolated, thermally denatured, and truncated forms of crystallin proteins (44–47). In our study, we identified and provided new evidence for an E3 ubiquitin ligase, RNF114 (48–50), that mediates and maintains the protein turnover and homeostasis of CRYAA in the lens. RNF114 appears to specifically recognize CRYAA as a substrate, and promoting its polyubiquitination and proteasomal degradation may be one of its key mechanisms.

We used 2 models of aggregated proteins: Y118D and WT CRYAA in a low-temperature model. Both can induce cataract formation and are effectively mitigated by RNF114. The WT CRYAA aggregates in the low-temperature model likely result from disrupted conformational balance or cellular responses to cold stress, while Y118D is a clinically validated cataract-associated CRYAA mutant protein. Notably, the aggregation mechanisms of these 2 proteins may differ. Although RNF114 effectively mitigates both Y118D and low-temperature WT CRYAA aggregates, they cannot be considered identical mechanisms. Under physiological conditions, endogenous CRYAA is soluble, and RNF114 does not significantly affect the levels of soluble CRYAA (as observed in GS iLECs in Figure 4, K and L). This selectivity may be influenced by its own structural characteristics and a variety of factors in the cellular environment. Typically, ubiquitination targets lysine residues accessible to E2 and E3 enzymes, with protein tertiary structure dictating lysine availability (51). Given that proteins may alter their conformation with temperature changes (52), the specifics of how these alterations affect α-crystallin’s ubiquitination at lower temperatures remain an area for future investigations. Additionally, we posit that protein modifications contribute to RNF114’s selective ubiquitination and subsequent clearance of CRYAA under varying conditions. Lens-specific E3 ubiquitin ligases selectively target and decompose crystallins exhibiting abnormal structures (53), thus preserving lens protein turnover and homeostasis.

We overexpressed exogenous GFP-tagged WT CRYAA and observed that overexpression of WT CRYAA resulted in some puncta formation (Figure 5F). However, RNF114’s role in promoting the clearance of these puncta was less evident compared with Y118D. In cells overexpressing Y118D, the degradation process was more pronounced and could be inhibited by proteasome inhibitors, suggesting that the aggregates formed by mutant CRYAA are primarily ubiquitinated and degraded mediated by RNF114. In contrast, the puncta formed by overexpressed WT CRYAA might follow a different formation mechanism, resulting in less effective degradation by RNF114. Since overexpression of WT CRYAA in LECs represents a non-physiological state, it is likely that, under such a stress condition, CRYAA, which is a molecular chaperone, may form large complexes with misfolded or unfolded client proteins, manifesting as “puncta.” Except for a small portion, possibly aggregates formed by accumulated CRYAA, the rest of the large complexes are likely not the target of RNF114. However, aggregates formed by endogenous CRYAA during low-temperature treatment or cataract formation showed a more significant response to RNF114.

Further experiments are warranted to accurately distinguish the different clearance mechanisms of overexpressed mutant CRYAA and WT CRYAA puncta, as well as the potential differences in aggregation mechanisms between Y118D and WT CRYAA under low-temperature conditions. Understanding these mechanisms is crucial for recognizing the role of the UPS and chaperone functions in maintaining the homeostasis of normal lens structural proteins.

To increase drug concentration within the lens, we engineered an RNF114 peptide fused with TAT to enhance its delivery into the lens; synthetic TAT-RNF114 has demonstrated efficacy in reversing the aggregation of mutant CRYAA and mitigating the irreversible lens opacification associated with cold-induced and oxidative cataracts in rats. Furthermore, this approach has proven effective in addressing related cataracts in H_2_O_2_-induced zebrafish models, both of which serve as validated models of cataractogenesis (54, 55). Thus, UPS-dependent E3 enzyme RNF114 as a key molecule in the prevention of lens protein aggregation points to a novel strategy for cataract prevention and treatment.

Taken together, these observations are likely to promote new research on the network of proteostasis maintenance in hibernators. The screening and design of drugs that stimulate specific protein degradation pathways can contribute to the precise regulation of protein stability and turnover, potentially leading to advances in proteostasis maintenance and the treatment of protein aggregation diseases.

## Methods

### Sex as a biological variable.

Our study was limited to male rats and GSs, leaving it uncertain whether the findings apply to females. Female rats’ estrous cycles may skew lens study results, unlike male rats’ stable hormones. This consistency is crucial when examining lens proteins during hibernation, where hormonal fluctuations could confound cold-induced changes.

### Cell culture and transfection.

GS iPSC line 1 and GS lenses were obtained from Wei Li, NIH, USA. For culture of GS iPSC line 1, the culture dishes were coated with 1% Matrigel, placed in a 37°C incubator for 30 minutes, and incubated as described previously.

For culture of GS iLECs and HLECs, F12 medium (Stem Cell Technologies) supplemented with 10% FBS (Thermo Fisher Scientific) and 1% penicillin-streptomycin was used, and the cells were incubated at 37°C in a 5% CO_2_ incubator. All cell lines tested negative for mycoplasma contamination.

For cell transfection, plasmids or siRNAs were lysed and encapsulated using Lipofectamine 3000 (Thermo Fisher Scientific) and transfected into GS iLECs and HLECs grown to 70% confluence according to the manufacturer’s instructions. After 24 hours of cell transfection, the medium was replaced with fresh medium, and subsequent experiments were performed. To assess silencing efficiency, quantitative PCR (qPCR) was used to quantify the expression of the target genes. Silencing efficiency greater than 80% was used as the standard for effective silencing. “RNF114 KD + RNF114 WT/ΔC” refers to the transfection of RNF114 WT/ΔC plasmid into cells where RNF114 has been knocked down. Specifically, RNF114 siRNA was first transfected into the cells, and after 24 hours, successful knockdown was verified, followed by the transfection of RNF114 WT/ΔC plasmid into the cells.

### GS lens cell differentiation in vitro.

The day before differentiation, GS iPSCs were replated on a Matrigel-covered Petri dish at 1 × 10^5^ cells/cm^2^, and an appropriate amount of KF (KSRF12) medium (Advanced DMEM [Thermo Fisher Scientific]/F12 with 15% KSR, 50 ng/mL bFGF, 1× NEAA, 100 μM 2-MA, 1× AA) was added. From days 1 to 5 (stage 1), the medium was replaced with NBD (N2B27 differentiation) medium (Advanced DMEM/F12 with 1% N2, 2% B27, 1× GlutaMAX [Thermo Fisher Scientific], 100 μM 2-MA, 1× AA). Subsequently, on days 6–9 (stage 2), differentiation was continued in BD (B27 differentiation) medium (Advanced DMEM/F12 with 2% B27, 1× GlutaMAX, 100 μM 2-MA, 1× AA) supplemented with 100 nM LDN193189 and 10 μM SB431542, and from day 10, the medium was replaced with FD (FBS differentiation) medium (Advanced DMEM/F12 with 8% FBS, 1× GlutaMAX, 100 μM 2-MA, 1× AA). On days 10–13 (stage 3), 20 ng/mL BMP4 and 10 μM SB431542 were added, while on days 14–21 (stage 4), 100 ng/mL bFGF and 20 ng/mL Wnt3a were added daily. At the end of stage 3, cells were digested and dispersed using TrypLE (Gibco), replated on Matrigel-coated dishes, and cultured for an additional week to mature into GS iLECs, which were supplemented with 10% FBS and 1% penicillin-streptomycin in F12 medium. Likewise, at the end of stage 3, the differentiated cells were mechanically passaged. Differentiating cells were scraped by scratching of the surface of the dish using an inoculating loop, and cell colonies were reseeded into new Matrigel-coated dishes for subsequent stage 4 differentiation.

### Hypothermia-rewarming model.

Rat lenses were obtained from postmortem male Sprague-Dawley rats (6 weeks old, 200 g; Shanghai SLAC Laboratory Animal Co. Ltd.). GS lenses were obtained from Wei Li, NIH, USA. For hypothermia treatment, GS/rat lenses or GS iLECs/HLECs were incubated at 4°C in Hibernate-A Medium (Thermo Fisher Scientific) supplemented with 10% FBS and 1% penicillin-streptomycin for 24 or 48 hours. After rewarming treatment, PBS washes were performed 3 times, and GS/rat lenses and GS iLECs/HLECs were incubated in prewarmed F12 medium supplemented with 10% FBS and 1% penicillin-streptomycin at 37°C. After incubation for 30 minutes, the cells were washed with PBS and placed in prewarmed extracellular solution (Beyotime) for observation or subsequent experiments.

### Liquid chromatography–tandem mass spectrometry proteomics.

The lenses of rats and GSs were isolated after hypothermia treatment for 8 hours or incubation at room temperature for 8 hours. The lens capsules were separated from the lens cortex after being washed in 4°C PBS, rapidly frozen in liquid nitrogen, and then stored at −80°C as samples for further proteomic analysis. Three biological replicates were examined for each experiment. The samples were lysed and digested using pressure cycling technology (PCT), labeled with TMT 10-plex label reagents (Thermo Fisher Scientific), and analyzed by liquid chromatography–tandem mass spectrometry (LC-MS/MS) with the Ultimate 3000 nanoLC-MS/MS system (Dionex LC Packings) coupled with a Q Exactive HF-X mass spectrometer (QE-HFX, Thermo Fisher Scientific) in data-dependent acquisition mode as described previously (56).

### MS data analysis and visualization.

The resultant MS data for GSs and rats were analyzed with Proteome Discoverer (version 2.4.1.15, Thermo Fisher Scientific) using a protein database composed of the Rodentia FASTA database (downloaded from UniProtKB on May 12, 2020, containing 26,850 reviewed protein sequences) and a protein database composed of the rat FASTA database (downloaded from UniProtKB on May 12, 2020, containing 9,949 reviewed protein sequences), respectively. The enzyme, static modification, and precursor ion mass tolerance parameters were set as described previously (57). The peptide-spectrum match allowed a 1% target false discovery rate (FDR) (strict) and a 5% target FDR (relaxed). Normalization was performed against the total peptide amount. The default setup was used for all other parameters. We used 2-tailed, 2-sided Student’s *t* tests to identify the differentially expressed proteins (DEPs) between 2 groups. DEPs were defined by thresholds of an adjusted *P* value less than 0.05 and a |log_2_ fold change| value greater than 0.25.

GO enrichment analysis of DEPs was performed with the Database for Annotation, Visualization and Integrated Discovery (DAVID 2021). The cutoff value was an FDR less than 0.01, and the results were visualized with R software (v3.6.1). Protein-protein interaction analysis was performed with STRING (v11.5), and the network was visualized with Cytoscape (v3.9.1; https://github.com/cytoscape/cytoscape/releases/3.9.1).

### Pull-down and MS experiments.

After 24 hours of hypothermia-rewarming treatment, GS iLECs were lysed in modified MYC lysis buffer as previously described. The cell lysates were centrifuged at 15,000*g* for 15 minutes at 4°C to remove intact cells. The supernatant was incubated with anti-CRYAA antibody–conjugated magnetic beads for 2 hours to pull down CRYAA. The beads were washed 4 times with lysis buffer and boiled at 100°C for 5 minutes. The samples were run on SDS-PAGE gels and then stained with Coomassie brilliant blue R250. Only the CRYAA band was retained, trypsinized, and subjected to MS. Individual proteins were identified using a Thermo Fisher Scientific Finnigan LTQ. The MS experiments and data processing were completed by the Shanghai Institute of Biochemistry and Cell Biology. All results are listed in Supplemental Table 4.

### Cataract assessment and lens transmittance measurement.

Isolated rat or GS lenses were placed in Petri dishes with extracellular solution and photographed with a Nikon SMZ18 microscope.

For lens transmittance measurement, a dish containing extracellular solution was placed on the bottom light source of the microscope. The untreated lens was placed in the center of the field of view, and image A was captured. Subsequently, the light source was turned off, and image B was captured. Image B was used to correct for ambient light interference. Afterward, the bottom light was turned on, the treated lens was placed directly above the bottom light source lamp, and image C was taken. The gray-scale values of the lens area were analyzed by ImageJ (NIH), and the transmittance was calculated according to the following formula:
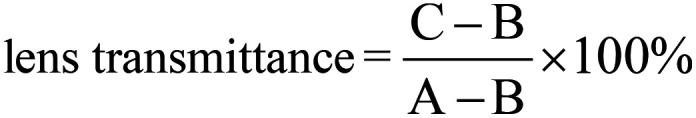


Cataract zebrafish were divided into 2 groups (4 fish in each group). The zebrafish in each group were anesthetized with 4% 3-aminobenzoic acid methyl ester and then placed in a 10 cm Petri dish, and the lenses of both eyes were photographed with a Nikon SMZ18 microscope. Then, one group was treated with TAT-RNF114 (100 μM) for 12 hours, and the other group served as the control group. Both groups of zebrafish were then anesthetized, and their lenses were photographed as described above. Transparency improvements were qualitatively assessed by visual analysis and graded using criteria adapted from LOCS III (58, 59): stage 0, clear lens; stage 1, loss of normal appearance of anterior, nuclear, and posterior lenses as well as prominence of *y*-suture line; stage 2, discrete anterior changes accompanied by distinct nuclear opacity; stage 3, increased involvement of nuclear and cortical regions of the lens accompanied by increasing opacity; stage 4, completely mature cataract involving the cortex and nucleus.

### Real-time qPCR analysis.

Each treated cell group was rinsed 3 times with PBS, and total RNA was extracted with an RNeasy Mini Kit (Qiagen). The RNA concentrations were quantified with a Nanodrop Spectrophotometer 1000 (Thermo Fisher Scientific), and the RNA was subsequently converted to cDNA with a reverse transcription kit (PrimeScriptRT Reagent Kit, TaKaRa). A TB Green Premix Ex Taq Kit (TaKaRa) and a 7500 Fast Real-Time PCR System (ABI) were used for real-time qPCR in 2 steps. The expression levels of target genes were normalized to those of β-actin and calculated as the mean ± SE using the 2^–ΔΔCt^ method, and statistical analysis was performed using a 2-tailed Student’s *t* test. The primers used for real-time qPCR are shown in Supplemental Table 2.

### Immunofluorescence staining and imaging.

Treated cells were rinsed with PBS and fixed with 4% paraformaldehyde (Sigma-Aldrich) for 15–30 minutes. Subsequently, the cells were treated with 0.5% Triton X-100 for 5 minutes and incubated with primary antibodies diluted in Antibody Dilution Buffer (A1800, Solarbio) at 4°C overnight. The cells were then incubated with an immunofluorescence secondary antibody (Invitrogen) for 1 hour at room temperature. The cells were treated with fluorescent mounting medium containing DAPI (Sigma-Aldrich). Confocal microscopy was performed using a Nikon A1 confocal microscope. ImageJ software (1.52a) was used to analyze the intensity of fluorescent spots. The antibodies are listed in Supplemental Table 3.

### Frozen sections.

After incubation with TAT-RNF114 (100 μM) for 30 minutes, isolated rat lenses were fixed in 4% paraformaldehyde, washed with PBS, permeabilized with 30% sucrose overnight, embedded in OCT compound (Tissue-Tek), and frozen at −80°C. A CM 1850 UV cryotome (Leica) was used to cut 5 μm thick sections of the coronal lens. For staining, sections were incubated in TNB blocking buffer (0.1 M Tris-HCl, 0.15 M NaCl, and 0.5% blocking reagent; PerkinElmer) for 30 minutes and incubated with primary antibodies in TNB at 4°C overnight. After 5 washes in PBS, sections were incubated with fluorochrome-conjugated secondary antibodies in TNB for 1 hour. The samples were mounted on coverslips with fluorescent mounting medium (Sigma-Aldrich) containing DAPI, and images were taken and analyzed as described above. The antibodies are listed in Supplemental Table 3.

### Co-IP and immunoblotting.

For co-IP assays, cells from each treatment group were lysed in modified MYC lysis buffer (MLB) (20 mM Tris-Cl, 200 mM NaCl, 10 mM NaF, 1 mM Na_3_V2O_4_ [Sigma-Aldrich, 450243], 1% NP-40 [Thermo Fisher Scientific, 85,124], 20 mM β-glycerophosphate [Sigma-Aldrich, G9422], and protease inhibitor [Complete Protease Inhibitor Cocktail, Roche, 04693116001], pH 7.5). Then, the samples were washed, bound to magnetic beads, separated, and quantified by SDS-PAGE. Western blotting with primary and secondary antibodies was performed to quantify the expression of target proteins in the samples and cell lysates as described above. The bands were visualized using Immobilon Western Chemiluminescent HRP Substrate (Millipore, Germany). The results were detected with a ChemiDoc Touch imaging system (Bio-Rad) and analyzed using ImageJ software (1.52a). The relative levels of all proteins were normalized to the levels of GAPDH. The antibodies are listed in Supplemental Table 3.

### Cell viability analysis.

A Cell Counting Kit-8 (Dojindo Molecular Technologies) was used according to the manufacturer’s protocol to detect the cytotoxicity of GS iLECs and HLECs in each treatment group. Twenty-four hours after transfection, 1 × 10^4^ cells/100 μL were added to each well of a 96-well plate (Thermo Fisher Scientific) and subjected to hypothermia treatment for 0–72 hours. Cell viability was then detected. Cells transfected with the empty plasmid were used as the control group (set to 1) to calculate the fold change.

### Flow cytometry analysis.

GS iPSCs and GS iLECs were digested and dispersed using TrypLE (Gibco), fixed with 4% paraformaldehyde (Sigma-Aldrich) for 15 minutes, and permeabilized with 0.5% Triton X-100 for 5 minutes. The cells were then incubated with CRYAA primary antibodies for 1 hour at 4°C followed by fluorochrome-conjugated secondary antibodies for 30 minutes at room temperature. Next, the cells were analyzed using a CytoFLEX LX and Cytoexpert software (Beckman).

### Live-cell imaging.

Twenty-four hours after transfection with plasmid or siRNA, the transfection efficiency of the cells was verified by confocal microscopy or qPCR. Cells were then subjected to hypothermia-rewarming for 24 or 48 hours followed by imaging with a Nikon A1 confocal microscope or a LEICA DMi8 inverted fluorescence microscope. Images of the first time point were taken, and the *xy* axis values of this field of view were recorded to ensure that subsequent images of the same field of view were taken at a given time point. At least 5 different well-focused cell areas were measured in each experimental group. Finally, the intensity of fluorescent spots was analyzed with ImageJ software (1.52a). Significant intracellular protein aggregates are defined as smaller, punctate, or granular aggregates within the cytoplasm that are clearly discernible. These broad, uniformly fluorescent zones are not classified as genuine intracellular aggregate foci and are thus excluded from our aggregation spot counts.

### Statistics.

All data are from at least 3 independent experiments and are expressed as the mean ± SD. Statistical analysis was performed by 2-tailed Student’s *t* test or ANOVA using GraphPad. *P* less than 0.05 was considered to indicate statistical significance.

### Study approval.

All animal procedures were approved by the University of Wisconsin–Oshkosh Institutional Animal Care and Use Committee and complied with the Animal Welfare Act (NIH/Department of Health and Human Services) and Association for Assessment and Accreditation of Laboratory Animal Care International (AAALAC) regulations. All animal experiments complied with the Vision and Ophthalmology Research Association Statement on the Use of Animals in Ophthalmology and Vision Research. All animals were handled according to the Laboratory Animal Care Administration of Zhejiang University. Zebrafish with H_2_O_2_-induced cataracts were obtained from Huaijin Guan, Nantong University, China (54), and all treatments were performed under the approval of the Animal Care and Use Committee of Nantong University. All experiments were performed in accordance with the *Guide for the Care and Use of Laboratory Animals* of the NIH (National Academies Press, 2011).

### Data availability.

All data associated with this study are presented in the paper or in the supplemental materials. Values for all data points in graphs are reported in the Supporting Data Values file.

## Author contributions

XS conceived the idea. XS and WL co-supervised the project. HY, KY, HA, FMNN, KJM, and JW designed and performed the experiments and analyzed the data. HY and XP drafted the manuscript. HY and JB took the zebrafish images. YH, YC, JZ, XX, and SW participated in the support of ubiquitin-proteasome system detection. All authors discussed the results and implications at all stages.

## Supplementary Material

Supplemental data

Unedited blot and gel images

Supporting data values

## Version 1 09/17/2024

Electronic publication
