# Supplementary material for: Reversible cold-induced lens opacity in a hibernator reveals a molecular target for treating cataracts
Source: J Clin Invest. 2024 Sep 17;134(18):e169666. doi: 10.1172/JCI169666 (PMC11405036; doi:10.1172/JCI169666)
Supplement: Supplemental data [file jci-134-169666-s209.pdf]

SUPPLEMENTARY MATERIAL

Figure S1. The degradation of aggregated αB-crystallin

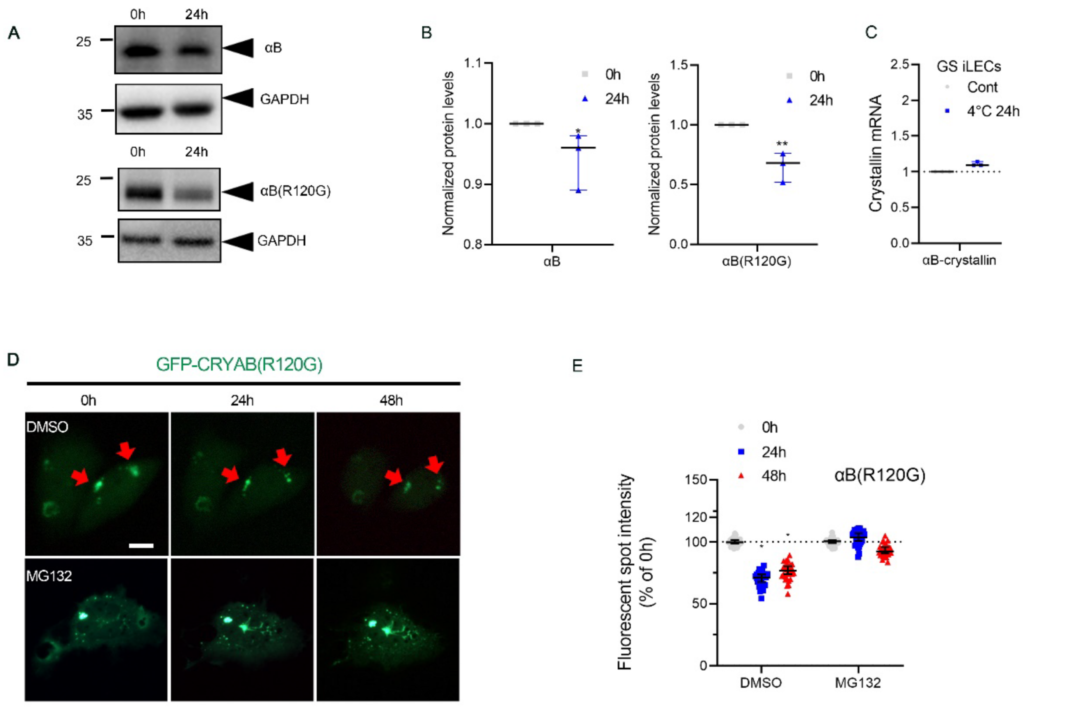

A. Immunoblotting was conducted to assess the expression levels of relevant proteins in GS iLECs after 24 hours of low-temperature rewarming. Endogenous CRYAB and exogenous CRYAB(R120G) mutant. **B.** Statistical representation of protein levels from Figure A. (n = 3 independent experiments) **C.** qPCR was employed to measure the expression levels of CRYAB mRNA in GS iLECs after 24 hours of low-temperature rewarming. (n = 3 independent experiments) **D.** Live-cell imaging was utilized to examine the intracellular localization of CRYAB(R120G) mutant in MG132-treated GS iLECs in the 24 and 48-hour low-temperature rewarming treatment groups and control group. DMSO was used as a control. Red arrows indicate typical degraded protein aggregates. Scale bar: 8 μm. **E.** Statistical analysis of fluorescence spot intensity from Figure D. (n = 3 independent experiments, > 5 cells per experiment) (All values are presented as mean ± SD, *P < 0.05, **P < 0.01, and ***P < 0.001).

Table S1. Reagents.

**Reagents**	**Catalog#**	**Supplier**
Matrigel hESC-Qualified Matrix	354277	Corning,USA
Fetal Bovine Serum	1099141C	Thermo,USA
Hibernate-A medium	A1247501	Thermo,USA
Accutase Cell DissociationReagent	A1110501	Thermo,USA
6-wellcellcultureplate	353046	Falcon,USA
B-27Supplement（50X）	17504044	Thermo,USA
AdvancedF12	16234010	Thermo.USA
GlutaMAXSupplement	35050061	Thermo,USA
Penicillin-Streptomycin	15140-122	Thermo,USA
Bovine serum albumin （BSA）	V900933	Sigma,USA
Triton X-100	T8787	Sigma,USA
Mounting Medium,antifading (with DAPI)	S2110	Solarbio,China
RNeasy Mini Kit	74104	Qiagen,Germany
PrimeScriptRT Reagent Kit	RR037B	TaKaRa,Japan
TB Green Premix Ex Taq Kit	RR071A	TaKaRa,Japan
Pierce BCA Protein Assay Kit	23227	Thermo,USA
ImmobilonWesternChemiluminescent HRP Substrate（ECL）	P90719	Millipore,Germany
Cell Counting Kit-8	CK04	Dojindo,Japan
DMEM/F12	36254	Stem Cell

		Technologies,Canada
Knockout Serum Replacement	A3181502	Thermo.USA
Non-Essential Amino Acids	11140-050	Thermo USA
L-Glutamine	25030-081	Thermo,USA
b-FGF	233-FB-025	R&D.USA
LDN-193189	S2618	Selleck,China
Recombinant Human BMP-4	314-BP	R&D.USA
Recombinant Human Wnt-3a	5036-WN	R&D.USA
Antibiotic-Antimycotic	15240062	ThermoFisher.USA
N-2(100X)	17502001	Gibco,USA
B-27 (50X)	17504044	Gibco,USA
Extracellular Solution	C0216	Beyotime,China
Human Noggin	120-10C	PeproTech,USA
BMP-7	120-03P	PeproTech,USA
2-Mercaptoethanol	21985023	Gibco,USA
SB431542	S1067	Selleck,China
Antibody Dilution Buffer	A1800	Solarbio,China

Table S2. Sequences of qPCR primers.

qPCR targetgenes	5'primer	3'primer
NANOG	GGAGCAATCAGACCTGGAACAAC	CTCCAAGACTGGCTATTCOAAGACT
TFAP2A	AGTACAAGGTCACAGTGGCG	TTGAGGGCCTCGGTGAGATA
PAX6	CAGATCTGCCACTTCCCCTG	TCACTCCGCTGTGACTGTTC
SIX1	AAGCCTAGGGAGGGGGTTAG	TTGAGCACGCTCTCGTTCTT
CRYAA	GGCATGAAGTGAATCCTGT	TGGTGGAGGAACATAGGTC
CRYAB	CCCCATGTCTGTCCTGAGC	AGAACTGGTCAAAGAGGCGG
CRYBB2	CTGAGGCCCATCAAAGTGGA	CTGTCCTTGTAGTCGCCCTT
GS RNF114	GACUUGUGGAGCACUGCAATT	UUGCAGUGCUCCACAAGUCTT
Human RNF114	AATGTTCCAAACCG	TTGCAGTGTTCCAC

Table S3. Antibodies.

Antibody	Catalog#	Supplier	Concentration
NANOG	sc-134218	Santa Cruz，USA	IF1:50
CRYAA	sc-28306	Santa Cruz，USA	IF1:50 WB1:200 FCM1:200
SIX1	12891	CST，USA	IF1:50
PAX6	sc-81649	Santa Cruz，USA	IF1:50
CRYAB	sc-137143	Santa Cruz，USA	IF1:200
CRYBB2	sc-376006	Santa Cruz，USA	IF1:200
TFAP2A	sc-12726	Santa Cruz，USA	IF1:50
ERK2	sc-514302	SantaCruz，USA	WB1:1000
P62	23214	CST，USA	WB1:1000
GADPH	sc-137179	SantaCruz，USA	WB1:1000
Ub	ab140601	Abcam，USA	WB1:1000
HA	ab18181	Abcam，USA	WB1:1000
RNF114	ab97303	Abcam，USA	WB1:1000
c-Myc	ab185655	Abcam，USA	WB1:1000
HIV1-Tat	MA1-71513	Invitrogen,USA	IF1:200
ALEXA FLUOR 488 GOAT	A10667	Invitrogen,USA	IF1:200
ALEXA FLUOR 555 DONKEY	A31572	Invitrogen,USA	IF1:200
ALEXA FLUOR 647 DONKEY	A31573	Invitrogen,USA	IF1:200

Table S4. Mass Spectrometry.

Control		Hypothermia-Rewarming treatment (4℃ 24 hours→37℃ 30 minutes)	
**Swiss-Prot ID**	**Description & Gene Symbol**	**Swiss-Prot ID**	**Description & Gene Symbol**
A0A287D744	RNA transcription, translation and transport factor protein OS=Ictidomys tridecemlineatus OX=43179 PE=3 SV=1	I3N0Y3	40S ribosomal protein S3 OS=Ictidomys tridecemlineatus OX=43179 GN=Rps3 PE=3 SV=1
I3M820	Cytokeratin-1 OS=Ictidomys tridecemlineatus OX=43179 GN=KRT1 PE=3 SV=2	A0A287D744	RNA transcription, translation and transport factor protein OS=Ictidomys tridecemlineatus OX=43179 PE=3 SV=1
I3M405	Lin-28 homologue A OS=Ictidomys tridecemlineatus OX=43179 GN=Lin28a PE=3 SV=2	I3MCU8	Small nuclear ribonucleoprotein polypeptide A' OS=Ictidomys tridecemlineatus OX=43179 GN=SNRPA1 PE=3 SV=1
I3N7V2	40S ribosomal protein S4 OS=Ictidomys tridecemlineatus OX=43179 GN=LOC101956897 PE=3 SV=2	I3M7N6	40S ribosomal protein S8 OS=Ictidomys tridecemlineatus OX=43179 GN=Rps8 PE=3 SV=1
I3MA46	Keratin 5 OS=Ictidomys tridecemlineatus OX=43179 GN=KRT5 PE=3 SV=2	I3N432	Protein-L-isoaspartate O-methyltransferase OS=Ictidomys tridecemlineatus OX=43179 GN=PCMT1 PE=3 SV=2
I3LZY6	Keratin 10 OS=Ictidomys tridecemlineatus OX=43179 GN=KRT10 PE=3 SV=2	I3MKR8	F-actin capping protein subunit β OS=Ictidomys tridecemlineatus OX=43179 GN=CAPZB PE=3 SV=2
I3N486	Heat shock 27 kDa protein OS=Ictidomys tridecemlineatus OX=43179 GN=HSPB1 PE=3 SV=1	I3N7V2	40S ribosomal protein S4 OS=Ictidomys tridecemlineatus OX=43179 GN=LOC101956897 PE=3 SV=2
I3N0Y3	40S ribosomal protein S3 OS=Ictidomys tridecemlineatus OX=43179 GN=Rps3 PE=3 SV=1	I3M820	Cytokeratin-1 OS=Ictidomys tridecemlineatus OX=43179 GN=KRT1 PE=3 SV=2
I3M7N6	40S ribosomal protein S8 OS=Ictidomys tridecemlineatus OX=43179 GN=Rps8 PE=3 SV=1	I3N486	Heat shock 27 kDa protein OS=Ictidomys tridecemlineatus OX=43179 GN=HSPB1 PE=3 SV=1
I3N432	Protein-L-isoaspartate O-methyltransferase OS=Ictidomys tridecemlineatus OX=43179 GN=PCMT1 PE=3 SV=2	I3MA46	Keratin 5 OS=Ictidomys tridecemlineatus OX=43179 GN=KRT5 PE=3 SV=2
A0A287CUU9	Keratin 15 OS=Ictidomys tridecemlineatus OX=43179 GN=KRT15 PE=3 SV=1	I3MI39	Ribosomal protein L10 OS=Ictidomys tridecemlineatus OX=43179 GN=Rpl10 PE=4 SV=2
I3MMC5	Contains KH RNA binding domain,	I3MJ79	Keratin 6A OS=Ictidomys

	signal transduction related 1 OS=Ictidomys tridecemlineatus OX=43179 GN=KHDRBS1 PE=3 SV=2		tridecemlineatus OX=43179 GN=Krt6a PE=3 SV=2
I3MIA6	60S ribosomal protein L13 OS=Ictidomys tridecemlineatus OX=43179 GN=RPL13 PE=3 SV=2	I3M4I1	Ribosomal protein L7 OS=Ictidomys tridecemlineatus OX=43179 GN=RPL7 PE=3 SV=2
I3MJ79	Keratin 6A OS=Ictidomys tridecemlineatus OX=43179 GN=Krt6a PE=3 SV=2	I3MNL1	Actin β OS=Ictidomys tridecemlineatus OX=43179 GN=ACTB PE=3 SV=1
I3M2S9	RNA binding protein, mRNA processing factor OS=Ictidomys tridecemlineatus OX=43179 GN=RBPMS PE=4 SV=2	I3LZY6	Keratin 10 OS=Ictidomys tridecemlineatus OX=43179 GN=KRT10 PE=3 SV=2
I3LX42	PRMT1 chromatin target OS=Ictidomys tridecemlineatus OX=43179 GN=CHTOP PE=4 SV=2	I3N206	Tyrosine 3-monooxygenase/tryptophan 5-monooxygenase activating protein zeta OS=Ictidomys tridecemlineatus OX=43179 GN=YWHAZ PE=3 SV=1
I3NHP5	Peroxiredoxin-1 OS=Ictidomys tridecemlineatus OX=43179 GN=PRDX1 PE=3 SV=1	I3NHB6	ADP/ATP translocase OS=Ictidomys tridecemlineatus OX=43179 GN=Slc25a5 PE=3 SV=1
I3MI39	Ribosomal protein L10 OS=Ictidomys tridecemlineatus OX=43179 GN=Rpl10 PE=4 SV=2	I3M405	Lin-28 homologue A OS=Ictidomys tridecemlineatus OX=43179 GN=Lin28a PE=3 SV=2
I3M3H5	Sodium/potassium transport ATPase subunit α OS=Ictidomys tridecemlineatus OX=43179 GN=Atp1a3 PE=3 SV=2	A0A287D3M1	40S ribosomal protein S4 OS=Ictidomys tridecemlineatus OX=43179 PE=3 SV=1
I3M4I1	Ribosomal protein L7 OS=Ictidomys tridecemlineatus OX=43179 GN=RPL7 PE=3 SV=2	I3M2S9	RNA binding protein, mRNA processing factor OS=Ictidomys tridecemlineatus OX=43179 GN=RBPMS PE=4 SV=2
I3MCU8	Small nuclear ribonucleoprotein polypeptide A' OS=Ictidomys tridecemlineatus OX=43179 GN=SNRPA1 PE=3 SV=1	I3LX42	PRMT1 chromatin target OS=Ictidomys tridecemlineatus OX=43179 GN=CHTOP PE=4 SV=2
I3N8X9	Triose phosphate isomerase OS=Ictidomys tridecemlineatus OX=43179 GN=Tpi1 PE=3 SV=2	I3N476	ADP/ATP translocase OS=Ictidomys tridecemlineatus OX=43179 GN=SLC25A4 PE=3 SV=1
A0A287D3M1	40S ribosomal protein S4 OS=Ictidomys tridecemlineatus OX=43179 PE=3 SV=1	I3MIA6	60S ribosomal protein L13 OS=Ictidomys tridecemlineatus OX=43179 GN=RPL13 PE=3 SV=2
I3MNL1	Actin β OS=Ictidomys tridecemlineatus OX=43179 GN=ACTB PE=3 SV=1	I3MMC5	Contains KH RNA binding domain, signal transduction related 1 OS=Ictidomys tridecemlineatus OX=43179 GN=KHDRBS1 PE=3 SV=2

I3NHB6	ADP/ATP translocase OS=Ictidomys tridecemlineatus OX=43179 GN=Slc25a5 PE=3 SV=1	I3M2Q7	60S ribosomal protein L7a OS=Ictidomys tridecemlineatus OX=43179 GN=RPL7A PE=3 SV=1
I3M9N5	LSM12 homologue OS=Ictidomys tridecemlineatus OX=43179 GN=LSM12 PE=3 SV=2	I3LZQ5	60S ribosomal protein L9 OS=Ictidomys tridecemlineatus OX=43179 GN=Rpl9 PE=3 SV=1
I3MYY0	60S ribosomal protein L14 OS=Ictidomys tridecemlineatus OX=43179 GN=Rpl14 PE=3 SV=2	I3N8X9	Triose phosphate isomerase OS=Ictidomys tridecemlineatus OX=43179 GN=Tpi1 PE=3 SV=2
I3N327	60S ribosomal protein L29 OS=Ictidomys tridecemlineatus OX=43179 GN=Rpl29 PE=3 SV=1	I3MIU3	Ribosomal protein L18 OS=Ictidomys tridecemlineatus OX=43179 GN=Rpl18 PE=3 SV=1
I3MIU3	Ribosomal protein L18 OS=Ictidomys tridecemlineatus OX=43179 GN=Rpl18 PE=3 SV=1	A0A287DFC3	60S ribosomal protein L13a OS=Ictidomys tridecemlineatus OX=43179 GN=Rpl13a PE=3 SV=1
I3ND60	Keratin 8 OS=Ictidomys tridecemlineatus OX=43179 GN=KRT8 PE=3 SV=1	I3N710	Ribosomal protein L10a OS=Ictidomys tridecemlineatus OX=43179 GN=Rpl10a PE=4 SV=2
I3NFU5	Cationic tryptase 3 OS=Ictidomys tridecemlineatus OX=43179 GN=LOC101966003 PE=4 SV=1	I3MGP9	Ribosomal protein L19 OS=Ictidomys tridecemlineatus OX=43179 GN=RPL19 PE=3 SV=2
A0A287CZ76	Uncharacterized protein OS=Ictidomys tridecemlineatus OX=43179 PE=4 SV=1	I3MYY0	60S ribosomal protein L14 OS=Ictidomys tridecemlineatus OX=43179 GN=Rpl14 PE=3 SV=2
A0A287CZQ4	Tubulin α chain OS=Ictidomys tridecemlineatus OX=43179 GN=LOC101970177 PE=3 SV=1	I3M2U6	Tyrosine 3-monooxygenase/tryptophan 5-monooxygenase activating protein β OS=Ictidomys tridecemlineatus OX=43179 GN=YWHAB PE=3 SV=1
I3MMW1	Keratin 16 OS=Ictidomys tridecemlineatus OX=43179 GN=KRT16 PE=3 SV=2	A0A287CZX3	Uncharacterized protein OS=Ictidomys tridecemlineatus OX=43179 PE=3 SV=1 ribosome
I3MA74	Elongation factor 1-α OS=Ictidomys tridecemlineatus OX=43179 GN=EEF1A1 PE=3 SV=1	I3NED7	TRASH domain-containing protein OS=Ictidomys tridecemlineatus OX=43179 PE=3 SV=1
I3N710	Ribosomal protein L10a OS=Ictidomys tridecemlineatus OX=43179 GN=Rpl10a PE=4 SV=2	I3NEJ6	40S ribosomal protein S9 OS=Ictidomys tridecemlineatus OX=43179 GN=RPS9 PE=3 SV=1
A0A287CRT9	Eukaryotic translation initiation factor 4E OS=Ictidomys tridecemlineatus OX=43179 GN=EIF4E PE=3 SV=1	I3MMW1	Keratin 16 OS=Ictidomys tridecemlineatus OX=43179 GN=KRT16 PE=3 SV=2
A0A287CVQ7	Cationic trypsin-3 OS=Ictidomys tridecemlineatus OX=43179 GN=LOC101966592 PE=4 SV=1	A0A287CZQ4	Tubulin α chain OS=Ictidomys tridecemlineatus OX=43179 GN=LOC101970177 PE=3 SV=1
I3MY31	GTP-binding nuclear protein Ran	A0A287CUU9	Keratin 15 OS=Ictidomys

	OS=Ictidomys tridecemlineatus OX=43179 GN=RAN PE=3 SV=2		tridecemlineatus OX=43179 GN=KRT15 PE=3 SV=1
I3N5Y0	Heterogeneous nuclear ribonucleoprotein H2 OS=Ictidomys tridecemlineatus OX=43179 GN=HNRNPH2 PE=4 SV=1	I3MNC5	Chromobox 5 OS=Ictidomys tridecemlineatus OX=43179 GN=CBX5 PE=4 SV=1
I3MMU3	IF rod domain protein OS=Ictidomys tridecemlineatus OX=43179 PE=3 SV=2	I3NCU1	Tubulin α chain OS=Ictidomys tridecemlineatus OX=43179 GN=Tuba1a PE=3 SV=2
I3MGP9	Ribosomal protein L19 OS=Ictidomys tridecemlineatus OX=43179 GN=RPL19 PE=3 SV=2	I3MHK9	ADP/ATP translocase OS=Ictidomys tridecemlineatus OX=43179 GN=Slc25a6 PE=3 SV=2
I3M2W9	Heat shock protein family A (Hsp70) member 8 OS=Ictidomys tridecemlineatus OX=43179 GN=HSPA8 PE=3 SV=1	I3NHP5	Peroxiredoxin-1 OS=Ictidomys tridecemlineatus OX=43179 GN=PRDX1 PE=3 SV=1
I3M806	Keratin 2 OS=Ictidomys tridecemlineatus OX=43179 GN=KRT2 PE=3 SV=2	I3MD45	Small nuclear ribonucleoprotein polypeptide B2 OS=Ictidomys tridecemlineatus OX=43179 GN=Snrpb2 PE=4 SV=1
I3N206	Tyrosine 3-monooxygenase/tryptophan 5-monooxygenase activating protein zeta OS=Ictidomys tridecemlineatus OX=43179 GN=YWHAZ PE=3 SV=1	I3N5Y0	Heterogeneous nuclear ribonucleoprotein H2 OS=Ictidomys tridecemlineatus OX=43179 GN=HNRNPH2 PE=4 SV=1
I3MD45	Small nuclear ribonucleoprotein polypeptide B2 OS=Ictidomys tridecemlineatus OX=43179 GN=Snrpb2 PE=4 SV=1	I3N327	60S ribosomal protein L29 OS=Ictidomys tridecemlineatus OX=43179 GN=Rpl29 PE=3 SV=1
I3N476	ADP/ATP translocase OS=Ictidomys tridecemlineatus OX=43179 GN=SLC25A4 PE=3 SV=1	I3M5L8	60S ribosomal protein L17 OS=Ictidomys tridecemlineatus OX=43179 GN=LOC101966012 PE=3 SV=2
A0A287CRW6	Serine- and arginine-rich splicing factor 9 OS=Ictidomys tridecemlineatus OX=43179 GN=SRSF9 PE=4 SV=1	A0A287CVQ7	Cationic trypsin-3 OS=Ictidomys tridecemlineatus OX=43179 GN=LOC101966592 PE=4 SV=1
I3N7M0	Histone H4 OS=Ictidomys tridecemlineatus OX=43179 GN=LOC106145262 PE=3 SV=1	I3N7M0	Histone H4 OS=Ictidomys tridecemlineatus OX=43179 GN=LOC106145262 PE=3 SV=1
I3M196	Mitochondrial Ribosomal Protein L19 OS=Ictidomys tridecemlineatus OX=43179 GN=MRPL19 PE=3 SV=2	I3MA74	Elongation factor 1-α OS=Ictidomys tridecemlineatus OX=43179 GN=EEF1A1 PE=3 SV=1
I3MYK3	Actin, cytoplasmic 1-like OS=Ictidomys tridecemlineatus OX=43179 GN=LOC101954496 PE=3 SV=2	I3M6R7	Cleavage and polyadenylation specificity factor subunit 5 OS=Ictidomys tridecemlineatus OX=43179 GN=NUDT21 PE=3 SV=1

I3NGT5	Keratin, Type I Cytoskeleton 42 OS=Ictidomys tridecemlineatus OX=43179 GN=LOC101959317 PE=3 SV=2	I3NCB7	Phosphoglycerate mutase OS=Ictidomys tridecemlineatus OX=43179 GN=PGAM1 PE=3 SV=1
I3M2Q7	60S ribosomal protein L7a OS=Ictidomys tridecemlineatus OX=43179 GN=RPL7A PE=3 SV=1	I3MMF9	Tyrosine 3-monooxygenase/tryptophan 5-monooxygenase activating protein γOS=Ictidomys tridecemlineatus OX=43179 GN=YWHAG PE=3 SV=1
I3MKI7	Keratin, type I epidermis Ha4 OS=Ictidomys tridecemlineatus OX=43179 GN=LOC101976426 PE=3 SV=2	I3ND60	Keratin 8 OS=Ictidomys tridecemlineatus OX=43179 GN=KRT8 PE=3 SV=1
I3NCT4	Inhibin OS=Ictidomys tridecemlineatus OX=43179 GN=Phb PE=3 SV=1	I3NCT4	Inhibin OS=Ictidomys tridecemlineatus OX=43179 GN=Phb PE=3 SV=1
I3LXE8	Amino acid transporter OS=Ictidomys tridecemlineatus OX=43179 GN=SLC1A2 PE=3 SV=2	I3MY31	GTP-binding nuclear protein Ran OS=Ictidomys tridecemlineatus OX=43179 GN=RAN PE=3 SV=2
I3N2A7	Mitochondrial Ribosomal Protein S26 OS=Ictidomys tridecemlineatus OX=43179 GN=MRPS26 PE=3 SV=1	I3M2W9	Heat shock protein family A (Hsp70) member 8 OS=Ictidomys tridecemlineatus OX=43179 GN=HSPA8 PE=3 SV=1
I3NCN5	Heat shock protein 90 alpha family class B member 1 OS=Ictidomys tridecemlineatus OX=43179 GN=HSP90AB1 PE=3 SV=1	A0A287CZ76	Uncharacterized protein OS=Ictidomys tridecemlineatus OX=43179 PE=4 SV=1 ribosome
I3MMF9	Tyrosine 3-monooxygenase/tryptophan 5-monooxygenase activating protein γOS=Ictidomys tridecemlineatus OX=43179 GN=YWHAG PE=3 SV=1	I3M6Y6	Ribosomal protein S5 OS=Ictidomys tridecemlineatus OX=43179 GN=RPS5 PE=3 SV=1
A0A287DAG1	Cleavage and polyadenylation specificity factor subunit 5 OS=Ictidomys tridecemlineatus OX=43179 GN=NUDT21 PE=3 SV=1	A0A287DB08	Serine- and arginine-rich splicing factor 10 OS=Ictidomys tridecemlineatus OX=43179 GN=SRSF10 PE=4 SV=1
I3MK72	60S ribosomal protein L8 OS=Ictidomys tridecemlineatus OX=43179 GN=RPL8 PE=3 SV=1	I3M806	Keratin 2 OS=Ictidomys tridecemlineatus OX=43179 GN=KRT2 PE=3 SV=2
I3M7X5	Keratin, Type II Cytoskeleton 73 OS=Ictidomys tridecemlineatus OX=43179 GN=KRT73 PE=3 SV=1	I3M5B9	Ribosomal protein L23a OS=Ictidomys tridecemlineatus OX=43179 GN=Rpl23a PE=3 SV=1
A0A287DFN3	F-actin capping protein subunit β OS=Ictidomys tridecemlineatus OX=43179 GN=CAPZB PE=3 SV=1	I3MYK3	Actin, cytoplasmic 1-like OS=Ictidomys tridecemlineatus OX=43179 GN=LOC101954496 PE=3 SV=2
I3MGS6	Ribosomal protein L15 OS=Ictidomys	I3M196	Mitochondrial Ribosomal Protein L19

	tridecemlineatus OX=43179 GN=RPL15 PE=3 SV=1		OS=Ictidomys tridecemlineatus OX=43179 GN=MRPL19 PE=3 SV=2
I3MTT4	Mitochondrial Ribosomal Protein L2 OS=Ictidomys tridecemlineatus OX=43179 GN=MRPL2 PE=4 SV=2	I3MKD9	Keratin, Type II Cytoskeleton 75 OS=Ictidomys tridecemlineatus OX=43179 GN=KRT75 PE=3 SV=2
I3N5J8	Tyrosine 3-monooxygenase/tryptophan 5-monooxygenase activating protein eta OS=Ictidomys tridecemlineatus OX=43179 GN=YWHAH PE=3 SV=1	I3NFU5	Cationic tryptase 3 OS=Ictidomys tridecemlineatus OX=43179 GN=LOC101966003 PE=4 SV=1
A0A287CS09	Cleavage and polyadenylation specificity factor subunit 4 OS=Ictidomys tridecemlineatus OX=43179 GN=CPSF4 PE=3 SV=1	I3NAL3	40S ribosomal protein S11 OS=Ictidomys tridecemlineatus OX=43179 GN=RPS11 PE=3 SV=9
I3M5L8	60S ribosomal protein L17 OS=Ictidomys tridecemlineatus OX=43179 GN=LOC101966012 PE=3 SV=2	I3MIX5	39S ribosomal protein L28, mitochondrial OS=Ictidomys tridecemlineatus OX=43179 GN=MRPL28 PE=3 SV=2
I3MYV7	SEC22 vesicular transport protein homologue B OS=Ictidomys tridecemlineatus OX=43179 GN=SEC22B PE=3 SV=1	A0A287CRT9	Eukaryotic translation initiation factor 4E OS=Ictidomys tridecemlineatus OX=43179 GN=EIF4E PE=3 SV=1
I3MQN1	Uncharacterized protein OS=Ictidomys tridecemlineatus OX=43179 PE=4 SV=2	I3M5F9	Tyrosine 3-monooxygenase/tryptophan 5-monooxygenase activating proteinεOS=Ictidomys tridecemlineatus OX=43179 GN=YWHAE PE=3 SV=2
A0A287DFC3	60S ribosomal protein L13a OS=Ictidomys tridecemlineatus OX=43179 GN=Rpl13a PE=3 SV=1	I3M9N5	LSM12 homologue OS=Ictidomys tridecemlineatus OX=43179 GN=LSM12 PE=3 SV=2
I3NCB7	Phosphoglycerate mutase OS=Ictidomys tridecemlineatus OX=43179 GN=PGAM1 PE=3 SV=1	I3M167	Solute carrier family 25 member 1 OS=Ictidomys tridecemlineatus OX=43179 GN=SLC25A1 PE=3 SV=2
I3M6Z6	H1.1 Linker histone, cluster member OS=Ictidomys tridecemlineatus OX=43179 GN=H1-1 PE=3 SV=1	I3N2A7	Mitochondrial Ribosomal Protein S26 OS=Ictidomys tridecemlineatus OX=43179 GN=MRPS26 PE=3 SV=1
A0A287DGB3	Keratin 35 OS=Ictidomys tridecemlineatus OX=43179 GN=KRT35 PE=3 SV=1	I3MK29	40S ribosomal protein S2 OS=Ictidomys tridecemlineatus OX=43179 GN=Rps2 PE=3 SV=2
I3M2A7	Myosin light chain 6B OS=Ictidomys tridecemlineatus OX=43179 GN=MYL6B PE=4 SV=2	I3MGS6	Ribosomal protein L15 OS=Ictidomys tridecemlineatus OX=43179 GN=RPL15 PE=3 SV=1
I3MIX5	39S ribosomal protein L28, mitochondrial OS=Ictidomys tridecemlineatus OX=43179 GN=MRPL28 PE=3 SV=2	I3MFF8	Poly(A) binding protein cytoplasmic 4 OS=Ictidomys tridecemlineatus OX=43179 GN=PABPC4 PE=3 SV=2
I3MTQ7	Ribosomal_L23eN domain protein	I3MEQ7	RNA binding protein, mRNA

	OS=Ictidomys tridecemlineatus OX=43179 PE=4 SV=2		processing factor 2 OS=Ictidomys tridecemlineatus OX=43179 GN=RBPMS2 PE=4 SV=2
I3MHT0	Heterogeneous nuclear ribonucleoprotein H3 OS=Ictidomys tridecemlineatus OX=43179 GN=HNRNPH3 PE=4 SV=2	I3MTT4	Mitochondrial Ribosomal Protein L2 OS=Ictidomys tridecemlineatus OX=43179 GN=MRPL2 PE=4 SV=2
I3MFF8	Poly(A) binding protein cytoplasmic 4 OS=Ictidomys tridecemlineatus OX=43179 GN=PABPC4 PE=3 SV=2	I3MKI7	Keratin, type I epidermis Ha4 OS=Ictidomys tridecemlineatus OX=43179 GN=LOC101976426 PE=3 SV=2
I3M2U6	Tyrosine 3-monooxygenase/tryptophan 5-monooxygenase activating protein β OS=Ictidomys tridecemlineatus OX=43179 GN=YWHAB PE=3 SV=1	I3MMU3	IF rod domain protein OS=Ictidomys tridecemlineatus OX=43179 PE=3 SV=2
I3NED7	TRASH domain-containing protein OS=Ictidomys tridecemlineatus OX=43179 PE=3 SV=1	A0A287CRW6	Serine- and arginine-rich splicing factor 9 OS=Ictidomys tridecemlineatus OX=43179 GN=SRSF9 PE=4 SV=1
I3MTJ9	39S ribosomal protein L16, mitochondrial OS=Ictidomys tridecemlineatus OX=43179 GN=MRPL16 PE=3 SV=2	I3M9T0	Keratin, Epidermis Type II Hb6 OS=Ictidomys tridecemlineatus OX=43179 GN=LOC106144876 PE=3 SV=1
I3N185	Phosphate carrier protein, mitochondrial OS=Ictidomys tridecemlineatus OX=43179 GN=SLC25A3 PE=3 SV=1	I3NF29	Keratin, Epidermis Type II Hb6 OS=Ictidomys tridecemlineatus OX=43179 GN=LOC101970545 PE=3 SV=2
I3MV02	RIBOOMAL_L9 domain protein OS=Ictidomys tridecemlineatus OX=43179 PE=3 SV=2	I3M2A7	Myosin light chain 6B OS=Ictidomys tridecemlineatus OX=43179 GN=MYL6B PE=4 SV=2
I3MEQ7	RNA binding protein, mRNA processing factor 2 OS=Ictidomys tridecemlineatus OX=43179 GN=RBPMS2 PE=4 SV=2	I3MN21	Family 168 members with sequence similarity A OS=Ictidomys tridecemlineatus OX=43179 GN=FAM168A PE=3 SV=1
I3MPB8	Mitochondrial Ribosomal Protein S7 OS=Ictidomys tridecemlineatus OX=43179 GN=MRPS7 PE=3 SV=1	I3MV02	RIBOOMAL_L9 domain protein OS=Ictidomys tridecemlineatus OX=43179 PE=3 SV=2
I3MIE7	2-Phosphate-D-glycerate hydrolase OS=Ictidomys tridecemlineatus OX=43179 GN=ENO1 PE=3 SV=1	I3MQN1	Uncharacterized protein OS=Ictidomys tridecemlineatus OX=43179 PE=4 SV=2
A0A287DGB4	Heterogeneous nuclear ribonucleoprotein D OS=Ictidomys tridecemlineatus OX=43179 GN=HNRNPD PE=4 SV=1	I3MZM6	Polyadenylation Binding Protein OS=Ictidomys tridecemlineatus OX=43179 GN=Pabpc1 PE=3 SV=2
I3M9Y0	Glyceraldehyde-3-phosphate dehydrogenase OS=Ictidomys tridecemlineatus OX=43179 GN=Gapdh	I3N5J8	Tyrosine 3-monooxygenase/tryptophan 5-monooxygenase activating protein eta OS=Ictidomys tridecemlineatus

	PE=3 SV=2		OX=43179 GN=YWHAH PE=3 SV=1
I3M2S5	Nuclear FMR1 interacting protein 2 OS=Ictidomys tridecemlineatus OX=43179 GN=Nufip2 PE=4 SV=2	I3N185	Phosphate carrier protein, mitochondrial OS=Ictidomys tridecemlineatus OX=43179 GN=SLC25A3 PE=3 SV=1
I3MYK8	Ig-like domain protein OS=Ictidomys tridecemlineatus OX=43179 PE=4 SV=2	I3MK72	60S ribosomal protein L8 OS=Ictidomys tridecemlineatus OX=43179 GN=RPL8 PE=3 SV=1
I3MM20	Heterogeneous nuclear ribonucleoprotein D-like OS=Ictidomys tridecemlineatus OX=43179 GN=HNRNPDL PE=4 SV=2	I3MLQ3	trans-2,3-enoyl-CoA reductase OS=Ictidomys tridecemlineatus OX=43179 GN=TECR PE=3 SV=2
I3NCR3	Heterogeneous nuclear ribonucleoprotein A3 OS=Ictidomys tridecemlineatus OX=43179 GN=HNRNPA3 PE=4 SV=1	I3NCQ7	Ribosomal protein L26 OS=Ictidomys tridecemlineatus OX=43179 GN=RPL26 PE=3 SV=1
I3MM78	40S ribosomal protein SA OS=Ictidomys tridecemlineatus OX=43179 GN=RPSA PE=3 SV=2	I3MUB2	40S ribosomal protein S3a OS=Ictidomys tridecemlineatus OX=43179 GN=Rps3a PE=3 SV=1
A0A287CVY7	Serine- and arginine-rich splicing factor 7 OS=Ictidomys tridecemlineatus OX=43179 GN=SRSF7 PE=4 SV=1	I3M4N6	60S ribosomal protein L40 OS=Ictidomys tridecemlineatus OX=43179 GN=Kxd1 PE=3 SV=1
A0A287CX53	Annexin OS=Ictidomys tridecemlineatus OX=43179 GN=ANXA2 PE=3 SV=1	I3NCD1	RAB7A, RAS oncogene family member OS=Ictidomys tridecemlineatus OX=43179 GN=RAB7A PE=4 SV=1
I3MAQ1	Anionic trypsin-2 OS=Ictidomys tridecemlineatus OX=43179 GN=LOC101960778 PE=4 SV=1	I3NCW3	Cationic trypsin 3-like OS=Ictidomys tridecemlineatus OX=43179 GN=LOC101966300 PE=4 SV=1
I3M2S0	ATP synthase subunit α OS=Ictidomys tridecemlineatus OX=43179 GN=ATP5F1A PE=3 SV=2	I3MV09	Tyrosine 3-monooxygenase/tryptophan 5-monooxygenase activating protein theta OS=Ictidomys tridecemlineatus OX=43179 GN=Ywhaq PE=3 SV=2
A0A287D536	Plaque globin OS=Ictidomys tridecemlineatus OX=43179 GN=JUP PE=3 SV=1	I3M7X5	Keratin, Type II Cytoskeleton 73 OS=Ictidomys tridecemlineatus OX=43179 GN=KRT73 PE=3 SV=1
I3N6D0	1,4-β-N-Acetyl muramidase C OS=Ictidomys tridecemlineatus OX=43179 GN=LYZ PE=3 SV=1	I3NCN5	Heat shock protein 90 alpha family class B member 1 OS=Ictidomys tridecemlineatus OX=43179 GN=HSP90AB1 PE=3 SV=1
I3M7F7	Heterogeneous nuclear ribonucleoprotein A2/B1 OS=Ictidomys tridecemlineatus OX=43179 GN=HNRNPA2B1 PE=4 SV=2	I3MTJ9	39S ribosomal protein L16, mitochondrial OS=Ictidomys tridecemlineatus OX=43179 GN=MRPL16 PE=3 SV=2
I3MHZ0	Proteasome subunit α type OS=Ictidomys tridecemlineatus OX=43179 GN=Psma6	I3MWD9	Poly(A) binding protein core 1 OS=Ictidomys tridecemlineatus

	PE=3 SV=2		OX=43179 GN=PABPN1 PE=4 SV=2
I3MGW6	Proton transport NAD(P)(+) Transhydrogenase OS=Ictidomys tridecemlineatus OX=43179 GN=NNT PE=3 SV=2	I3MAQ1	Anionic trypsin-2 OS=Ictidomys tridecemlineatus OX=43179 GN=LOC101960778 PE=4 SV=1
I3N654	40S ribosomal protein S6 OS=Ictidomys tridecemlineatus OX=43179 PE=3 SV=2	I3MHZ0	Proteasome subunit α type OS=Ictidomys tridecemlineatus OX=43179 GN=Psma6 PE=3 SV=2
I3M167	Solute carrier family 25 member 1 OS=Ictidomys tridecemlineatus OX=43179 GN=SLC25A1 PE=3 SV=2	A0A0H3WGB9	40S ribosomal protein S6 OS=Ictidomys tridecemlineatus OX=43179 GN=rps6 PE=2 SV=1
I3NEJ6	40S ribosomal protein S9 OS=Ictidomys tridecemlineatus OX=43179 GN=RPS9 PE=3 SV=1	A0A287D6W8	Small nuclear ribonucleoprotein-related protein OS=Ictidomys tridecemlineatus OX=43179 GN=SNRPB PE=3 SV=1
A0A287D509	Nucleoside diphosphate kinase OS=Ictidomys tridecemlineatus OX=43179 GN=NME1 PE=3 SV=1	A0A287D509	Nucleoside diphosphate kinase OS=Ictidomys tridecemlineatus OX=43179 GN=NME1 PE=3 SV=1
A0A287DCJ4	60S ribosomal protein L21 OS=Ictidomys tridecemlineatus OX=43179 PE=3 SV=1	I3MHT0	Heterogeneous nuclear ribonucleoprotein H3 OS=Ictidomys tridecemlineatus OX=43179 GN=HNRNPH3 PE=4 SV=2
I3MM74	Calmodulin OS=Ictidomys tridecemlineatus OX=43179 GN=CNN3 PE=3 SV=2	I3M7G6	RNA binding motif protein X-linked OS=Ictidomys tridecemlineatus OX=43179 GN=Rbmx PE=4 SV=1
I3MY03	Cofilin, non-muscle isoform OS=Ictidomys tridecemlineatus OX=43179 GN=CFL1 PE=3 SV=2	I3M179	Myosin light chain 4 OS=Ictidomys tridecemlineatus OX=43179 GN=MYL4 PE=4 SV=2
I3MNC5	Chromobox 5 OS=Ictidomys tridecemlineatus OX=43179 GN=CBX5 PE=4 SV=1	A0A287CVD0	Keratin, Epidermis Type I Ha1 OS=Ictidomys tridecemlineatus OX=43179 GN=LOC101976712 PE=3 SV=1
I3LZM6	Perredoxin 4 OS=Ictidomys tridecemlineatus OX=43179 GN=PRDX4 PE=4 SV=2	I3NAA7	Histone H2B OS=Ictidomys tridecemlineatus OX=43179 GN=LOC101967584 PE=3 SV=1
I3NCW3	Cationic trypsin 3-like OS=Ictidomys tridecemlineatus OX=43179 GN=LOC101966300 PE=4 SV=1	I3MYV7	SEC22 vesicular transport protein homologue B OS=Ictidomys tridecemlineatus OX=43179 GN=SEC22B PE=3 SV=1
I3MWD9	Poly(A) binding protein core 1 OS=Ictidomys tridecemlineatus OX=43179 GN=PABPN1 PE=4 SV=2	A0A287DD77	Heterogeneous nuclear ribonucleoprotein A/B OS=Ictidomys tridecemlineatus OX=43179 GN=HNRNPAB PE=4 SV=1
I3MGJ2	Transgelin OS=Ictidomys tridecemlineatus OX=43179	I3N0W7	Mitochondrial Ribosomal Protein S34 OS=Ictidomys tridecemlineatus

	GN=TAGLN PE=3 SV=1		OX=43179 GN=MRPS34 PE=4 SV=1
A0A287DD77	Heterogeneous nuclear ribonucleoprotein A/B OS=Ictidomys tridecemlineatus OX=43179 GN=HNRNPAB PE=4 SV=1	I3MCZ4	ELAV-like protein OS=Ictidomys tridecemlineatus OX=43179 GN=ELAVL1 PE=3 SV=2
I3N5B8	Tubulin beta chain OS=Ictidomys tridecemlineatus OX=43179 GN=TUBB2A PE=3 SV=1	I3NGT5	Keratin, Type I Cytoskeleton 42 OS=Ictidomys tridecemlineatus OX=43179 GN=LOC101959317 PE=3 SV=2
I3MVI3	40S ribosomal protein S25 OS=Ictidomys tridecemlineatus OX=43179 GN=RPS25 PE=3 SV=1	I3MJS6	Secretion-related Ras-related GTPase 1A OS=Ictidomys tridecemlineatus OX=43179 GN=SAR1A PE=3 SV=1
I3MAD4	Mitochondrial ribosomal protein L24 OS=Ictidomys tridecemlineatus OX=43179 GN=Mrpl24 PE=3 SV=1	I3MMB4	RAB6B, RAS oncogene family member OS=Ictidomys tridecemlineatus OX=43179 GN=RAB6B PE=4 SV=2
I3M4N6	60S ribosomal protein L40 OS=Ictidomys tridecemlineatus OX=43179 GN=Kxd1 PE=3 SV=1	I3M6Z6	H1.1 Linker histone, cluster member OS=Ictidomys tridecemlineatus OX=43179 GN=H1-1 PE=3 SV=1
I3MYK9	Heterogeneous nuclear ribonucleoprotein F OS=Ictidomys tridecemlineatus OX=43179 GN=Hnrnpf PE=4 SV=1	A0A287CS09	Cleavage and polyadenylation specificity factor subunit 4 OS=Ictidomys tridecemlineatus OX=43179 GN=CPSF4 PE=3 SV=1
I3M7G6	RNA binding motif protein X-linked OS=Ictidomys tridecemlineatus OX=43179 GN=Rbmx PE=4 SV=1	I3MAE6	60S ribosomal protein L18a OS=Ictidomys tridecemlineatus OX=43179 GN=RPL18A PE=3 SV=2
A0A287D5T5	Peptidylprolyl cis-trans isomerase OS=Ictidomys tridecemlineatus OX=43179 GN=Ppia PE=3 SV=1	I3MAD4	Mitochondrial ribosomal protein L24 OS=Ictidomys tridecemlineatus OX=43179 GN=Mrpl24 PE=3 SV=1
i3MX94	DIRAS family GTPase 2 OS=Ictidomys tridecemlineatus OX=43179 GN=DIRAS2 PE=4 SV=1	I3MM20	Heterogeneous nuclear ribonucleoprotein D-like OS=Ictidomys tridecemlineatus OX=43179 GN=HNRNPDL PE=4 SV=2
I3MUB2	40S ribosomal protein S3a OS=Ictidomys tridecemlineatus OX=43179 GN=Rps3a PE=3 SV=1	I3M624	Elongation factor 1-β OS=Ictidomys tridecemlineatus OX=43179 GN=EEF1B2 PE=3 SV=1
I3MPF5	Keratin 78 OS=Ictidomys tridecemlineatus OX=43179 GN=KRT78 PE=3 SV=2	I3MUG9	Histone H2B OS=Ictidomys tridecemlineatus OX=43179 GN=H2BC5 PE=3 SV=2
I3N7W4	Profilin OS=Ictidomys tridecemlineatus OX=43179 GN=PFN1 PE=3 SV=1	A0A287DGB3	Keratin 35 OS=Ictidomys tridecemlineatus OX=43179 GN=KRT35 PE=3 SV=1
I3MMH6	enoyl-CoA hydratase, short chain 1 OS=Ictidomys tridecemlineatus OX=43179 GN=ECHS1 PE=3 SV=2	I3NCR3	Heterogeneous nuclear ribonucleoprotein A3 OS=Ictidomys tridecemlineatus OX=43179

			GN=HNRNPA3 PE=4 SV=1
I3MHK9	ADP/ATP translocase OS=Ictidomys tridecemlineatus OX=43179 GN=Slc25a6 PE=3 SV=2	I3M8D9	Mitochondrial Ribosomal Protein S23 OS=Ictidomys tridecemlineatus OX=43179 GN=MRPS23 PE=3 SV=2
I3MK29	40S ribosomal protein S2 OS=Ictidomys tridecemlineatus OX=43179 GN=Rps2 PE=3 SV=2	A0A287CVY7	Serine- and arginine-rich splicing factor 7 OS=Ictidomys tridecemlineatus OX=43179 GN=SRSF7 PE=4 SV=1
I3NFZ3	Histone H2A OS=Ictidomys tridecemlineatus OX=43179 GN=H2AC20 PE=3 SV=1	A0A287DFM0	DnaJ Heat shock protein family (Hsp40) member B6 OS=Ictidomys tridecemlineatus OX=43179 GN=Dnajb6 PE=4 SV=1
I3NCD1	RAB7A, RAS oncogene family member OS=Ictidomys tridecemlineatus OX=43179 GN=RAB7A PE=4 SV=1	A0A287DCJ4	60S ribosomal protein L21 OS=Ictidomys tridecemlineatus OX=43179 PE=3 SV=1
I3MN21	Family 168 members with sequence similarity A OS=Ictidomys tridecemlineatus OX=43179 GN=FAM168A PE=3 SV=1	I3M5X7	Transmembrane protein 33 OS=Ictidomys tridecemlineatus OX=43179 GN=TMEM33 PE=3 SV=2
I3MV09	Tyrosine 3-monooxygenase/tryptophan 5-monooxygenase activating protein theta OS=Ictidomys tridecemlineatus OX=43179 GN=Ywhaq PE=3 SV=2	A0A287D9A7	RAB1A, RAS oncogene family member OS=Ictidomys tridecemlineatus OX=43179 GN=Rab1a PE=4 SV=1
I3M2E2	40S ribosomal protein S19 OS=Ictidomys tridecemlineatus OX=43179 GN=RPS19 PE=3 SV=2	i3MXE6	RAB10, RAS oncogene family member OS=Ictidomys tridecemlineatus OX=43179 GN=RAB10 PE=4 SV=1
i3MXE6	RAB10, RAS oncogene family member OS=Ictidomys tridecemlineatus OX=43179 GN=RAB10 PE=4 SV=1	I3M9Y0	Glyceraldehyde-3-phosphate dehydrogenase OS=Ictidomys tridecemlineatus OX=43179 GN=Gapdh PE=3 SV=2
I3M0D5	40S ribosomal protein S18 OS=Ictidomys tridecemlineatus OX=43179 GN=Rps18 PE=3 SV=2	A0A287D7N9	Vimentin OS=Ictidomys tridecemlineatus OX=43179 GN=VIM PE=3 SV=1
I3MNC2	Dolichol-phosphate Mannosyltransferase Subunit 1 OS=Ictidomys tridecemlineatus OX=43179 GN=Dpm1 PE=3 SV=1	I3NFZ3	Histone H2A OS=Ictidomys tridecemlineatus OX=43179 GN=H2AC20 PE=3 SV=1
I3MGB5	60S ribosomal protein L31 OS=Ictidomys tridecemlineatus OX=43179 GN=RPL31 PE=3 SV=1	A0A287CZU6	Proteasome subunit α type OS=Ictidomys tridecemlineatus OX=43179 GN=PSMA5 PE=3 SV=1
		I3LWC6	Mitochondrial Ribosomal Protein L45 OS=Ictidomys tridecemlineatus OX=43179 GN=Mrpl45 PE=4 SV=2
		A0A287DGB4	Heterogeneous nuclear ribonucleoprotein D OS=Ictidomys tridecemlineatus OX=43179

	GN=HNRNPD PE=4 SV=1
A0A287D1F4	Tropomyosin 1 OS=Ictidomys tridecemlineatus OX=43179 GN=TPM1 PE=3 SV=1
I3MPE0	Peroxiredoxin-6 OS=Ictidomys tridecemlineatus OX=43179 GN=PRDX6 PE=3 SV=1
I3NEV3	40S ribosomal protein S7 OS=Ictidomys tridecemlineatus OX=43179 GN=RPS7 PE=3 SV=2
I3MVI3	40S ribosomal protein S25 OS=Ictidomys tridecemlineatus OX=43179 GN=RPS25 PE=3 SV=1
I3MM74	Calmodulin OS=Ictidomys tridecemlineatus OX=43179 GN=CNN3 PE=3 SV=2
I3MGW6	Proton transport NAD(P)(+) Transhydrogenase OS=Ictidomys tridecemlineatus OX=43179 GN=NNT PE=3 SV=2
I3MPB8	Mitochondrial Ribosomal Protein S7 OS=Ictidomys tridecemlineatus OX=43179 GN=MRPS7 PE=3 SV=1
I3MRY1	RRM domain protein OS=Ictidomys tridecemlineatus OX=43179 PE=4 SV=1
I3NCG3	Histone H3 OS=Ictidomys tridecemlineatus OX=43179 GN=LOC101963257 PE=3 SV=1
I3M7F7	Heterogeneous nuclear ribonucleoprotein A2/B1 OS=Ictidomys tridecemlineatus OX=43179 GN=HNRNPA2B1 PE=4 SV=2
I3MH39	60S acidic ribosomal protein P0 OS=Ictidomys tridecemlineatus OX=43179 GN=Rplp0 PE=3 SV=1
I3MQA9	Rho GDP dissociation inhibitor α OS=Ictidomys tridecemlineatus OX=43179 GN=ARHGDIA PE=3 SV=1
A0A287D764	Uncharacterized protein OS=Ictidomys tridecemlineatus OX=43179 PE=4 SV=1

I3M882	RAB5C, RAS oncogene family member OS=Ictidomys tridecemlineatus OX=43179 GN=RAB5C PE=4 SV=2
I3MNI4	Pre-mRNA splicing factor SPF27 OS=Ictidomys tridecemlineatus OX=43179 GN=BCAS2 PE=3 SV=1
I3LW01	Y-box binding protein 1 OS=Ictidomys tridecemlineatus OX=43179 GN=YBX1 PE=4 SV=2
I3LZC6	RAS-related OS=Ictidomys tridecemlineatus OX=43179 GN=RRAS PE=4 SV=1
I3MRS2	Succinate--CoA ligase [ADP/GDP forming] subunit alpha, mitochondrial OS=Ictidomys tridecemlineatus OX=43179 GN=SUCLG1 PE=3 SV=1
I3MPT4	γ-glutamyl cyclotransferase OS=Ictidomys tridecemlineatus OX=43179 GN=Ggct PE=4 SV=2
I3ML71	Proteasome subunit alpha type OS=Ictidomys tridecemlineatus OX=43179 GN=PSMA3 PE=3 SV=1
I3M2S0	ATP synthase subunit α OS=Ictidomys tridecemlineatus OX=43179 GN=ATP5F1A PE=3 SV=2
I3MRH9	39S ribosomal protein L59, mitochondrial OS=Ictidomys tridecemlineatus OX=43179 GN=GADD45GIP1 PE=3 SV=1
I3MYK8	Ig-like domain protein OS=Ictidomys tridecemlineatus OX=43179 PE=4 SV=2
I3LZM6	Perredoxin 4 OS=Ictidomys tridecemlineatus OX=43179 GN=PRDX4 PE=4 SV=2
I3MGJ2	Transgelin OS=Ictidomys tridecemlineatus OX=43179 GN=TAGLN PE=3 SV=1
I3MAH1	Transmembrane serine protease 13 OS=Ictidomys tridecemlineatus OX=43179 GN=TMPRSS13 PE=4 SV=2
I3MAL8	60S ribosomal protein L35

	OS=Ictidomys tridecemlineatus OX=43179 GN=RPL35 PE=3 SV=2
A0A287DCG1	Solute carrier family 25 members 10 OS=Ictidomys tridecemlineatus OX=43179 GN=Slc25a10 PE=3 SV=1
I3M6B3	H/ACA ribonucleoprotein complex subunit OS=Ictidomys tridecemlineatus OX=43179 GN=GAR1 PE=3 SV=2
I3M5L5	Zinc finger protein 326 OS=Ictidomys tridecemlineatus OX=43179 GN=ZNF326 PE=3 SV=2
A0A287D5T5	Peptidylprolyl cis-trans isomerase OS=Ictidomys tridecemlineatus OX=43179 GN=Ppia PE=3 SV=1
I3M9V0	Sodium/potassium transport ATPase subunit α OS=Ictidomys tridecemlineatus OX=43179 GN=ATP1A2 PE=3 SV=1
I3MMV7	S-methyl-5'-thioadenosine phosphorylase OS=Ictidomys tridecemlineatus OX=43179 GN=MTAP PE=3 SV=2
A0A287D536	Plaque globin OS=Ictidomys tridecemlineatus OX=43179 GN=JUP PE=3 SV=1
I3MDH3	60S ribosomal protein L6 OS=Ictidomys tridecemlineatus OX=43179 GN=RPL6 PE=3 SV=1
A0A287DEC1	Ribosomal protein S16 OS=Ictidomys tridecemlineatus OX=43179 GN=RPS16 PE=3 SV=1
I3M679	Hydroxysteroid 17-beta Dehydrogenase 10 OS=Ictidomys tridecemlineatus OX=43179 GN=HSD17B10 PE=3 SV=2
I3MGU2	Solute carrier family 25 members 11 OS=Ictidomys tridecemlineatus OX=43179 GN=SLC25A11 PE=3 SV=1
I3MMH6	enoyl-CoA hydratase, short chain 1 OS=Ictidomys tridecemlineatus OX=43179 GN=ECHS1 PE=3 SV=2
I3M0D5	40S ribosomal protein S18 OS=Ictidomys tridecemlineatus

	OX=43179 GN=Rps18 PE=3 SV=2
I3M186	DNA-guided RNA polymerase II subunit E OS=Ictidomys tridecemlineatus OX=43179 GN=POLR2E PE=3 SV=1
A0A287DF60	60S ribosomal protein L27a OS=Ictidomys tridecemlineatus OX=43179 GN=Rpl27a PE=3 SV=1
I3M2E2	40S ribosomal protein S19 OS=Ictidomys tridecemlineatus OX=43179 GN=RPS19 PE=3 SV=2
I3NDF7	40S ribosomal protein S23 OS=Ictidomys tridecemlineatus OX=43179 GN=Rps23 PE=3 SV=2
I3M1J1	Adenosylhomocysteinase OS=Ictidomys tridecemlineatus OX=43179 GN=AHCY PE=3 SV=1
I3MY77	Heart and neural crest derivatives express 1 OS=Ictidomys tridecemlineatus OX=43179 GN=HAND1 PE=4 SV=1
A0A287DCN5	Serine/arginine rich splicing factor 1 OS=Ictidomys tridecemlineatus OX=43179 GN=SRSF1 PE=3 SV=1
I3M2S5	Nuclear FMR1 interacting protein 2 OS=Ictidomys tridecemlineatus OX=43179 GN=Nufip2 PE=4 SV=2
I3N2A1	60S ribosomal protein L27 OS=Ictidomys tridecemlineatus OX=43179 GN=Rpl27 PE=3 SV=1
I3MI38	28S ribosomal protein S18-2, mitochondrial OS=Ictidomys tridecemlineatus OX=43179 GN=MRPS18B PE=3 SV=2
I3MVG7	GST class-pi OS=Ictidomys tridecemlineatus OX=43179 GN=GSTP1 PE=3 SV=2
I3MI28	Elongation factor Tu OS=Ictidomys tridecemlineatus OX=43179 GN=TUFM PE=3 SV=1
I3MFR9	Proteasome subunit β OS=Ictidomys tridecemlineatus OX=43179 GN=PSMB1 PE=3 SV=2
I3N498	Isopentenyl diphosphate

	delta-isomerase OS=Ictidomys tridecemlineatus OX=43179 GN=LOC101970547 PE=3 SV=1
I3M911	Activated C kinase 1 receptor OS=Ictidomys tridecemlineatus OX=43179 GN=RACK1 PE=4 SV=1
I3MJ90	Proteasome 20S subunit α 7 OS=Ictidomys tridecemlineatus OX=43179 GN=PSMA7 PE=3 SV=1
A0A287D7I5	Proteasome subunit β OS=Ictidomys tridecemlineatus OX=43179 PE=3 SV=1
I3MLS1	Proteasome subunit β OS=Ictidomys tridecemlineatus OX=43179 GN=PSMB7 PE=3 SV=2
**A0A287CXY5**	**RINGtype E3ubiquitin transferase OS=Ictidomys tridecemlineatus OX=43179 GN=RNF114 PE=4 SV=1**
I3M350	Methyltransferase-like 26 OS=Ictidomys tridecemlineatus OX=43179 GN=METTL26 PE=3 SV=1
I3MCQ1	40S ribosomal protein S13 OS=Ictidomys tridecemlineatus OX=43179 GN=RPS13 PE=3 SV=1
A0A287DBG7	Desmoplakin OS=Ictidomys tridecemlineatus OX=43179 GN=DSP PE=3 SV=1
I3N7W4	Profilin OS=Ictidomys tridecemlineatus OX=43179 GN=PFN1 PE=3 SV=1
I3M6D9	Succinate dehydrogenase [ubiquinone] iron-sulfur subunit, mitochondrial OS=Ictidomys tridecemlineatus OX=43179 GN=SDHB PE=3 SV=2
A0A287D120	Bet1 Golgi vesicle membrane transporter-like OS=Ictidomys tridecemlineatus OX=43179 GN=Bet1l PE=4 SV=1
I3N9M0	Optineurin OS=Ictidomys tridecemlineatus OX=43179 GN=LOC101955860 PE=4 SV=2
I3MC11	ATP synthase peripheral stem subunit OSCP OS=Ictidomys tridecemlineatus OX=43179 GN=Atp5po PE=3 SV=2

I3LWH9	[Histone H3]-Trimethyl-L-Lysine(4) Demethylase OS=Ictidomys tridecemlineatus OX=43179 GN=KDM5B PE=3 SV=1
I3N5R7	H(+)-transport second sector ATPase OS=Ictidomys tridecemlineatus OX=43179 PE=3 SV=2
i3MX94	DIRAS family GTPase 2 OS=Ictidomys tridecemlineatus OX=43179 GN=DIRAS2 PE=4 SV=1
A0A287D9J0	Pyruvate Kinase OS=Ictidomys tridecemlineatus OX=43179 GN=PKLR PE=3 SV=1
I3MC38	Trifunctional purine biosynthesis protein adenosine-3 OS=Ictidomys tridecemlineatus OX=43179 GN=GART PE=3 SV=1
I3M3R6	Solute carrier family 25 members 39 OS=Ictidomys tridecemlineatus OX=43179 GN=SLC25A39 PE=3 SV=2
I3N893	Taste receptor type 2 OS=Ictidomys tridecemlineatus OX=43179 PE=3 SV=2
I3MA33	ATP synthase subunit b OS=Ictidomys tridecemlineatus OX=43179 GN=Atp5pb PE=3 SV=2
I3LX11	28S ribosomal protein S15, mitochondrial OS=Ictidomys tridecemlineatus OX=43179 GN=MRPS15 PE=3 SV=1
I3N2E7	Chromobox 6 OS=Ictidomys tridecemlineatus OX=43179 GN=CBX6 PE=4 SV=2
I3MDR7	Malignant fibrous histiocytoma amplified sequence 1 OS=Ictidomys tridecemlineatus OX=43179 GN=Mfhas1 PE=4 SV=2
I3MKJ3	Diacylglycerol lipase α OS=Ictidomys tridecemlineatus OX=43179 GN=DAGLA PE=4 SV=1
A0A287CYQ7	STING ER export protein OS=Ictidomys tridecemlineatus OX=43179 GN=CXorf56 PE=3 SV=1

I3MIE7	2-Phosphate-D-glycerate hydrolase OS=Ictidomys tridecemlineatus OX=43179 GN=ENO1 PE=3 SV=1
I3MQ02	Keratin 18 OS=Ictidomys tridecemlineatus OX=43179 GN=KRT18 PE=3 SV=2
T1YVM8	Hemoglobin beta (fragment) OS=Ictidomys tridecemlineatus OX=43179 GN=HBB PE=2 SV=1
I3NAE5	60S ribosomal protein L36a-like OS=Ictidomys tridecemlineatus OX=43179 GN=LOC101965105 PE=3 SV=2
I3N3A1	RAB2A, RAS oncogene family member OS=Ictidomys tridecemlineatus OX=43179 GN=RAB2A PE=4 SV=2
I3M9C9	Glutathione S-transferase OS=Ictidomys tridecemlineatus OX=43179 GN=LOC101960145 PE=3 SV=1
A0A287DCS3	Histone Acetyltransferase OS=Ictidomys tridecemlineatus OX=43179 GN=KAT6B PE=3 SV=1
I3N5B8	Tubulin beta chain OS=Ictidomys tridecemlineatus OX=43179 GN=TUBB2A PE=3 SV=1
I3LZM4	Zinc finger matrin-type 2 OS=Ictidomys tridecemlineatus OX=43179 GN=ZMAT2 PE=4 SV=1

Following a 24-hour period of low-temperature re-warming, CRYAA was precipitated from the lysates of GS iLECs. Subsequently, it underwent SDS-PAGE and was subjected to mass spectrometry for the isolation and identification of individual proteins. The control group consisted of non-low-temperature treated GS iLECs, with three samples in each group. The following abbreviations have been utilized: OS (Organism), OX (NCBI_TaxID), GN (Gene Name), PE (Protein Existence), and SV (Sequence Version).
