# Supplementary material for: Reversible cold-induced lens opacity in a hibernator reveals a molecular target for treating cataracts
Source: J Clin Invest. 2024 Sep 17;134(18):e169666. doi: 10.1172/JCI169666 (PMC11405036; doi:10.1172/JCI169666)
Supplement: Unedited blot and gel images [file jci-134-169666-s210.pdf]

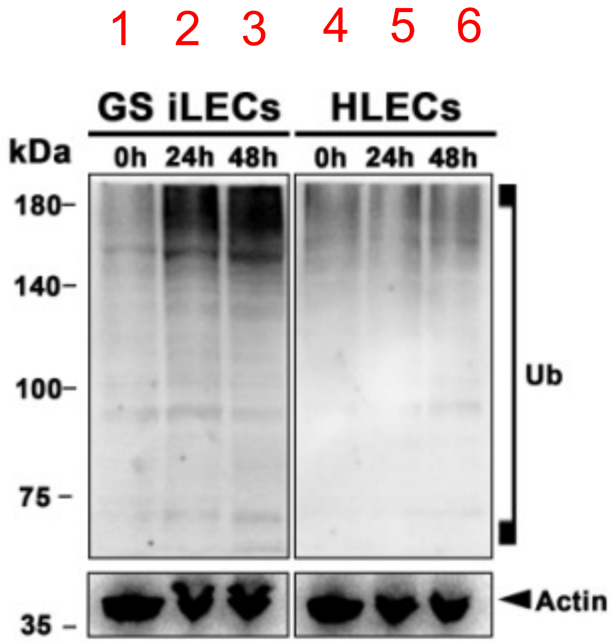
1 2 3 4 5 6
GS iLECs
HLECs
kDa
0h 24h 48h 0h 24h 48h
180
140
100
75
35
Ub
Actin

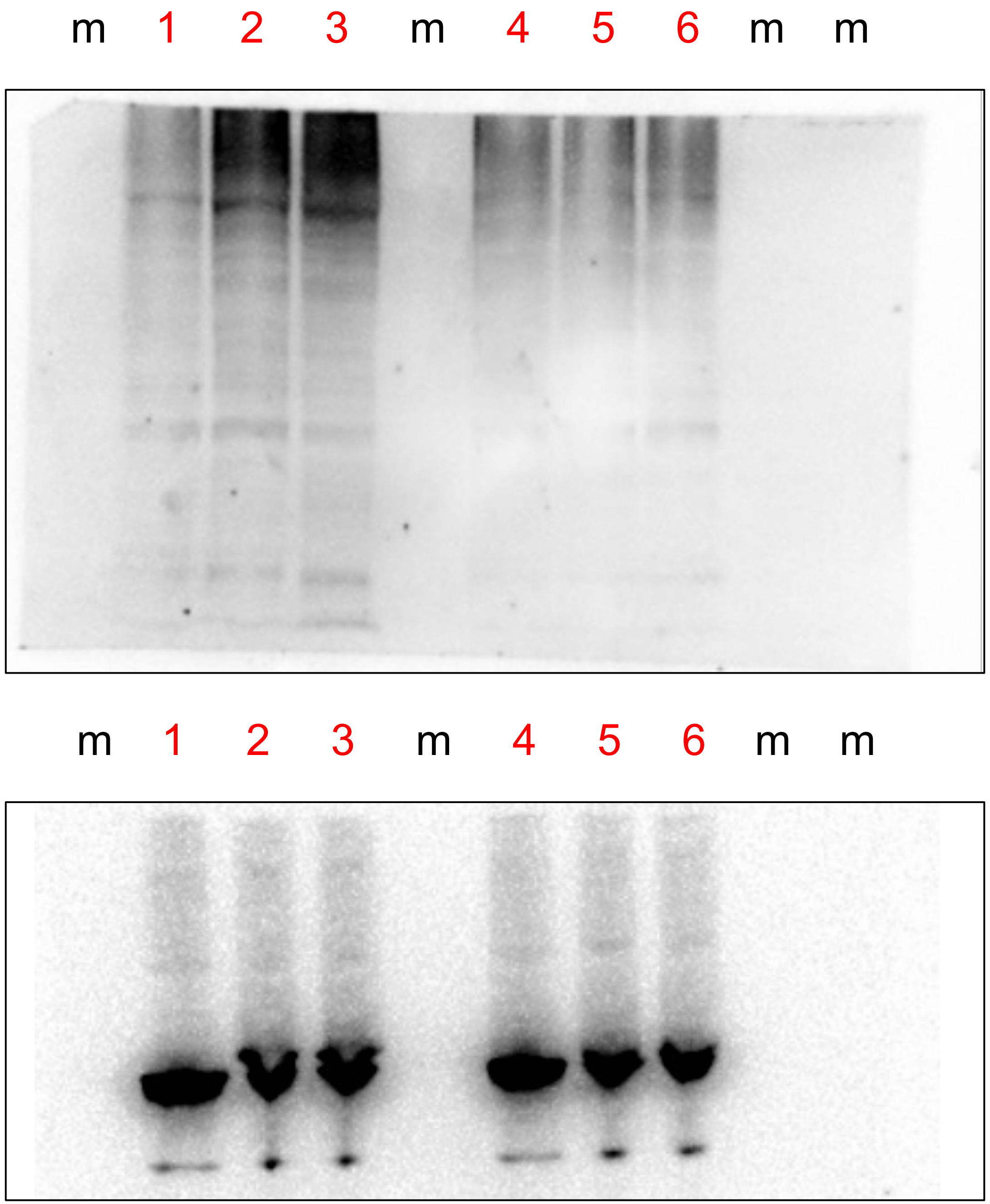
m 1 2 3 m 4 5 6 m m
m 1 2 3 m 4 5 6 m m

Fig. 3. C

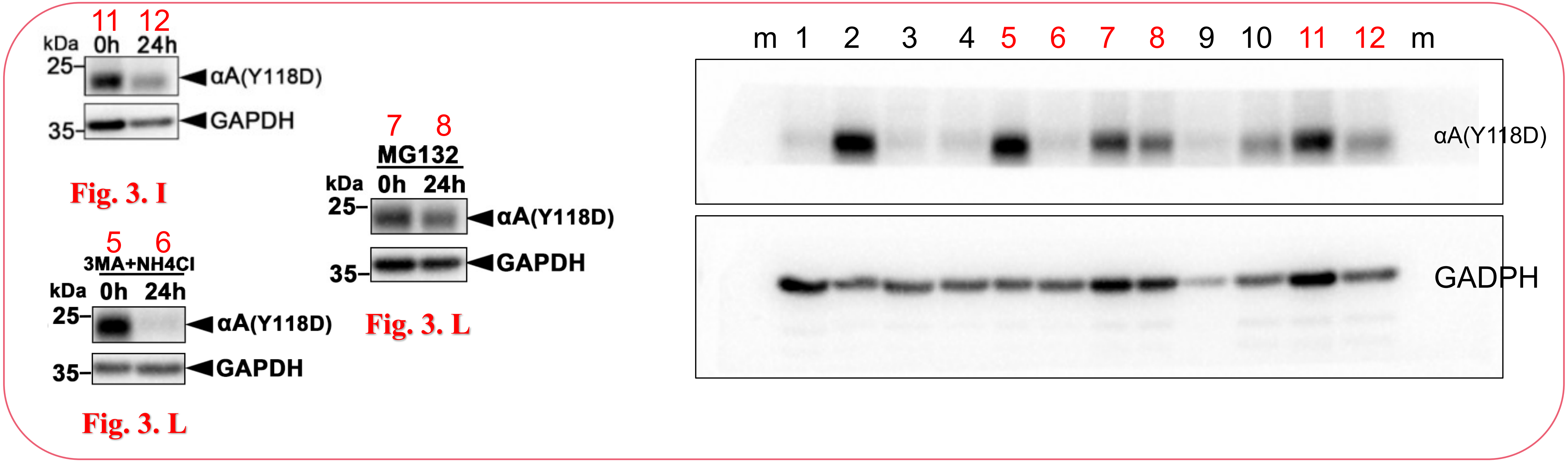

Fig. 3. I
Fig. 3. L
Fig. 3. L

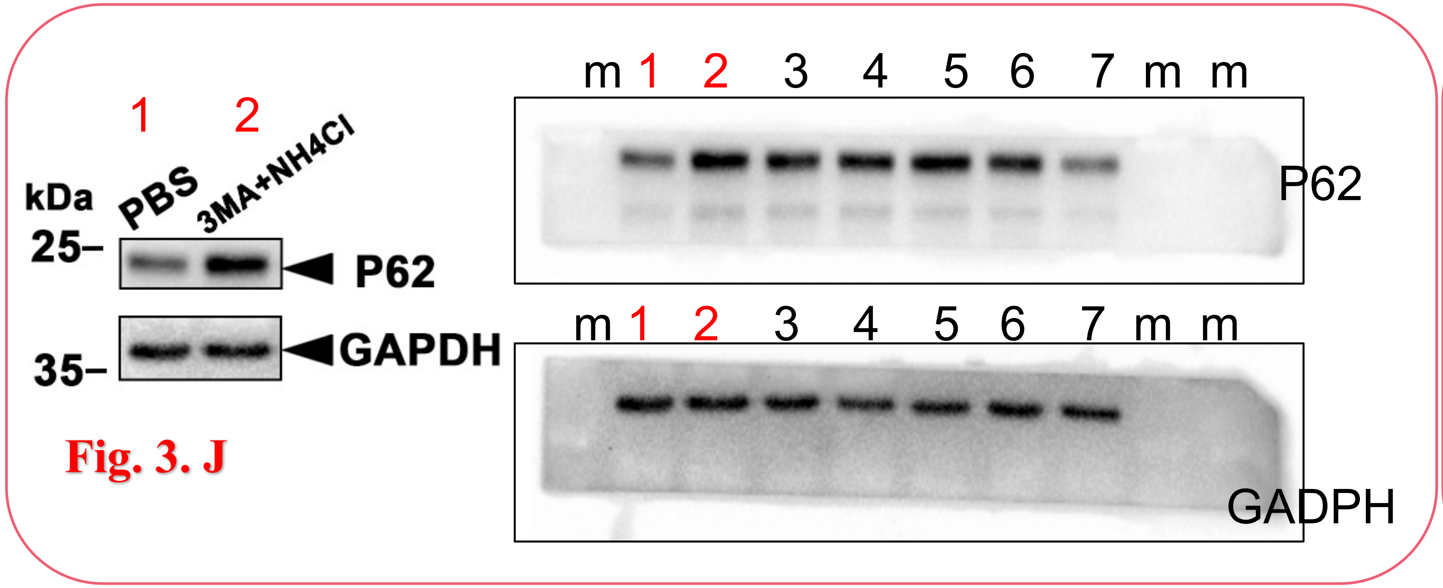

Fig. 3. J

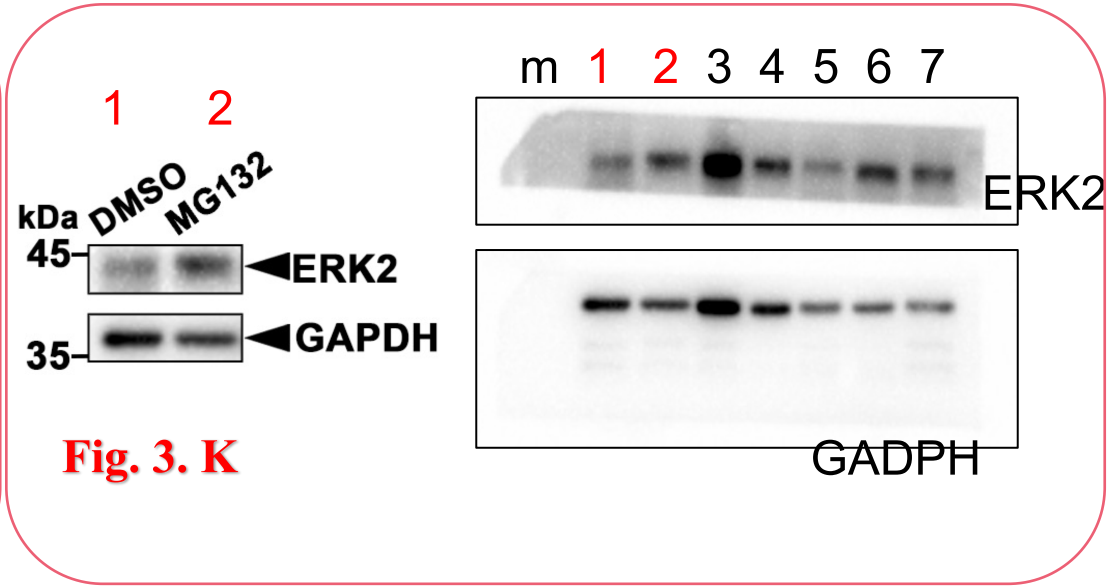

Fig. 3. K

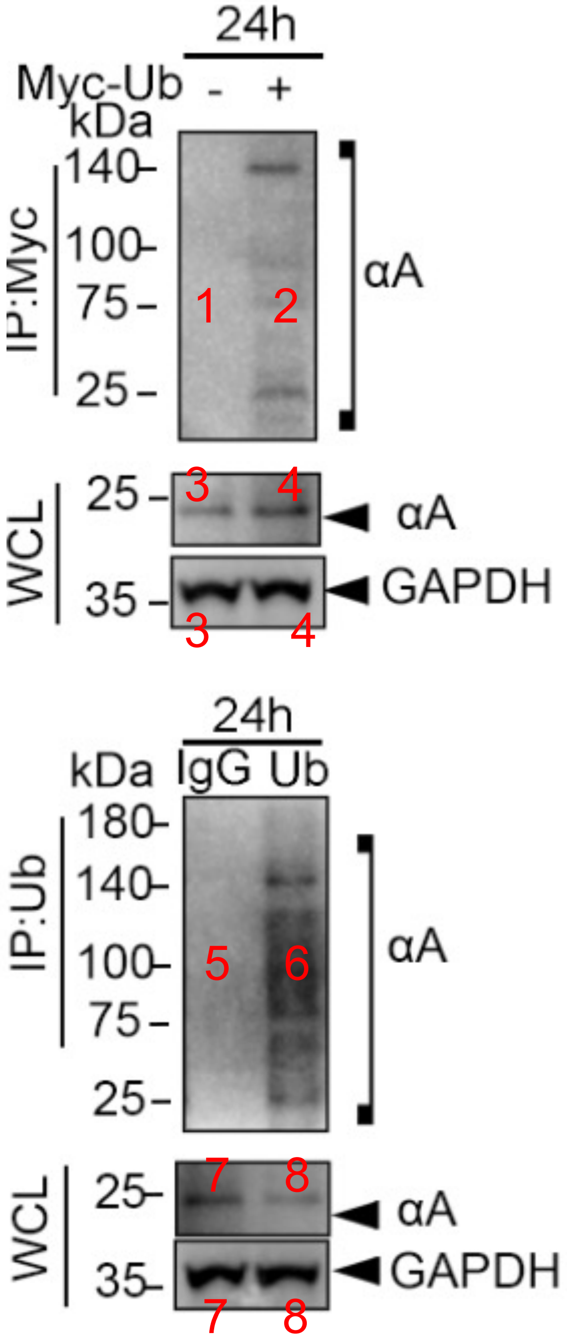
24h
Myc-Ub - +
kDa
140–
100–
75 –
25 –
IP:Myc
1 2
αA
WCL
25 –
35 –
3 4
αA
GAPDH
3 4
24h
kDa IgG Ub
180–
140–
100–
75 –
25–
IP:Ub
5 6
αA
WCL
25–
35–
7 8
αA
GAPDH
7 8

Fig. 4. C

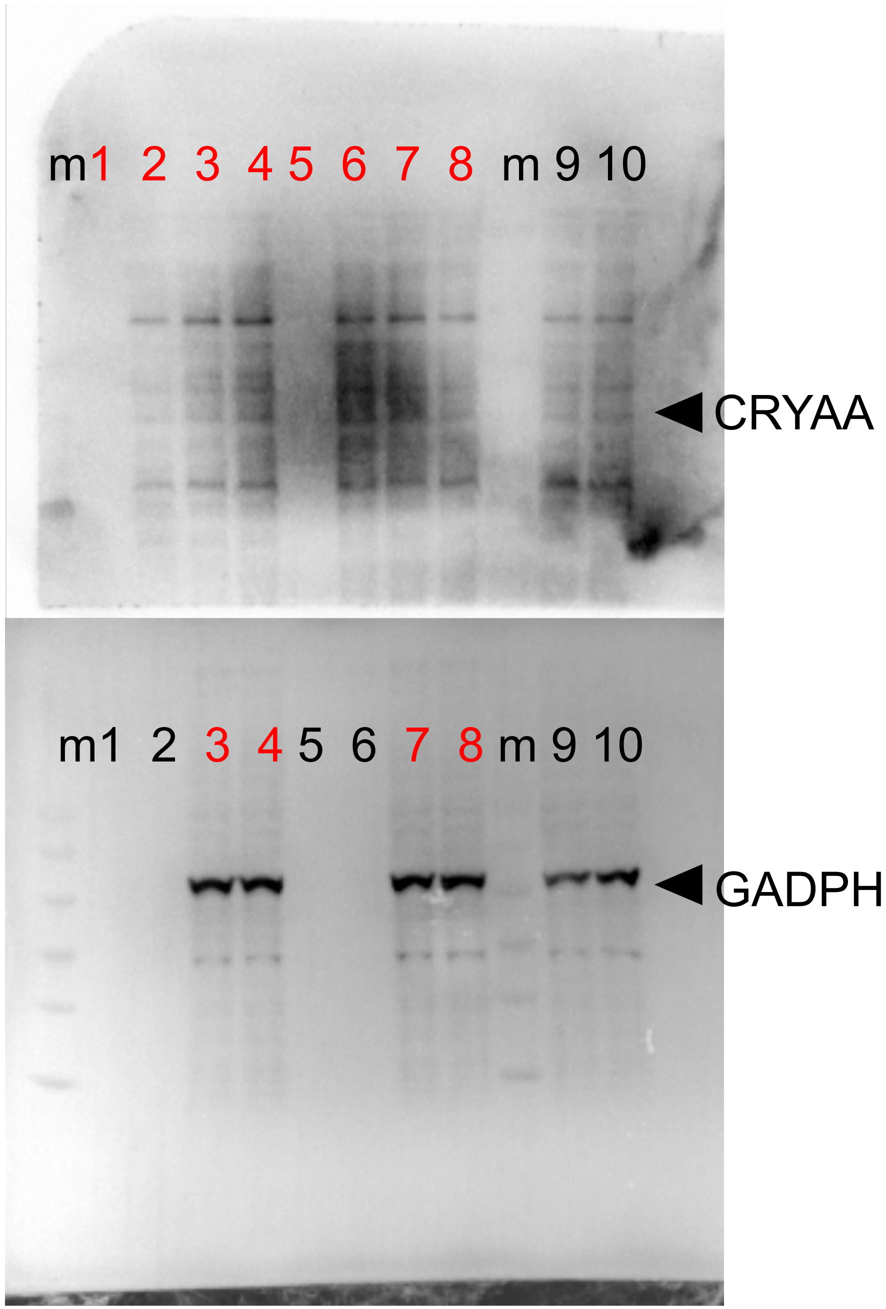
m1 2 3 4 5 6 7 8 m 9 10
CRYAA
m1 2 3 4 5 6 7 8 m 9 10
GADPH

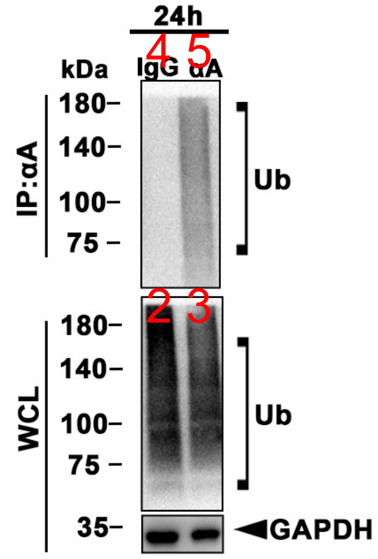
24h
4 5
kDa IgG αA
180–
140–
100–
75 –
IP:αA
Ub
2 3
180–
140–
100–
75 –
35–
WCL
Ub
GAPDH

Fig. 4. D

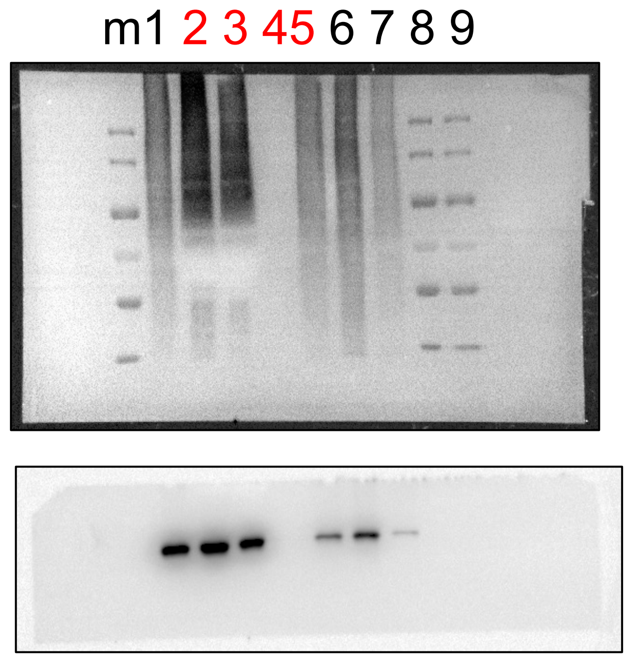
m1 2 3 45 6 7 8 9

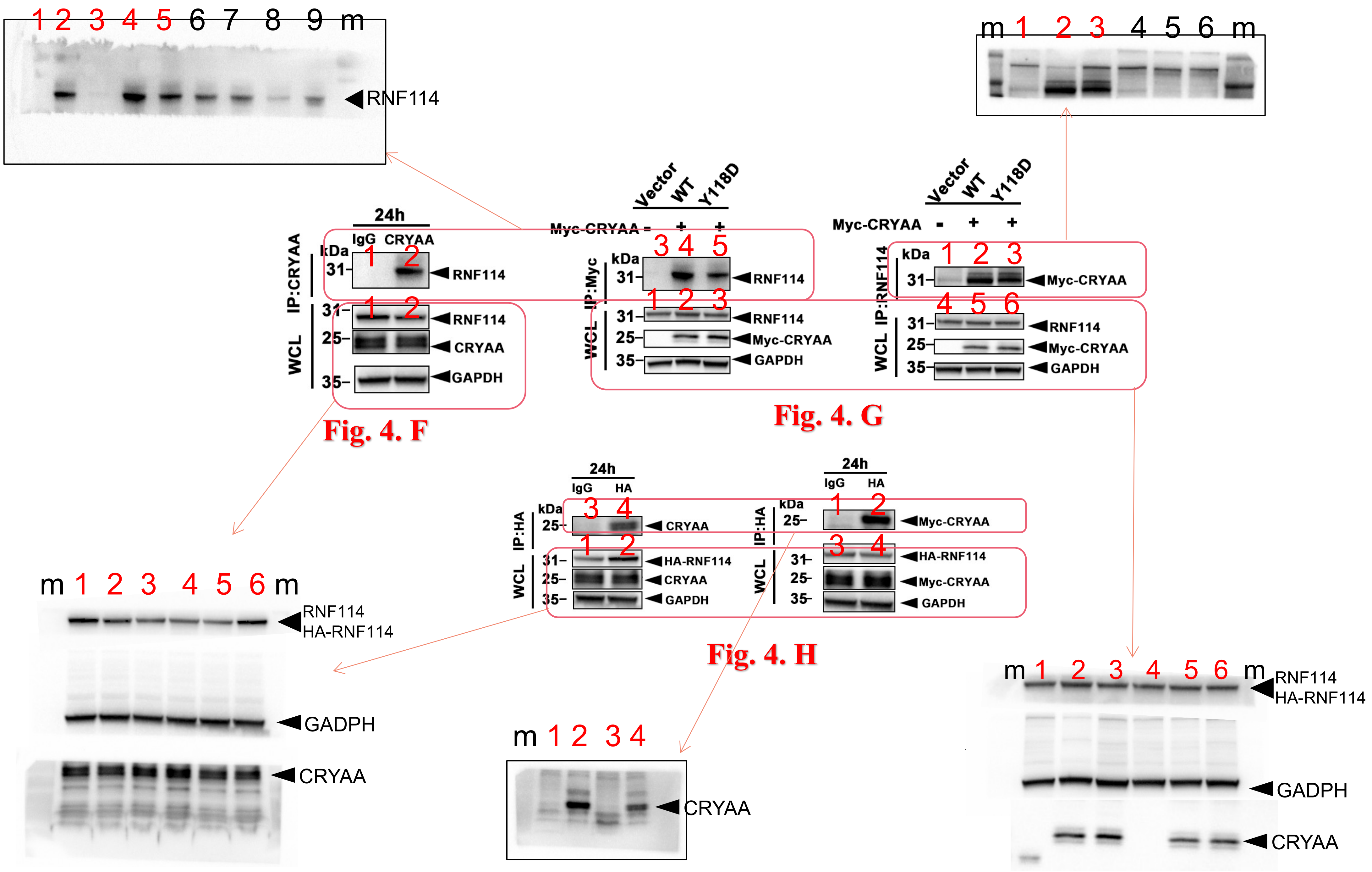

RNF114
IP:CRYAA
WCL
24h
IgG CRYAA
kDa
31-
25-
35-
RNF114
CRYAA
GAPDH
Fig. 4. F
Vector WT Y118D
Myc-CRYAA - + +
IP:Myc
Myc-CRYAA
Fig. 4. G
IP:RNF114
HA-RNF114
GADPH
IgG HA
IP:HA
Fig. 4. H

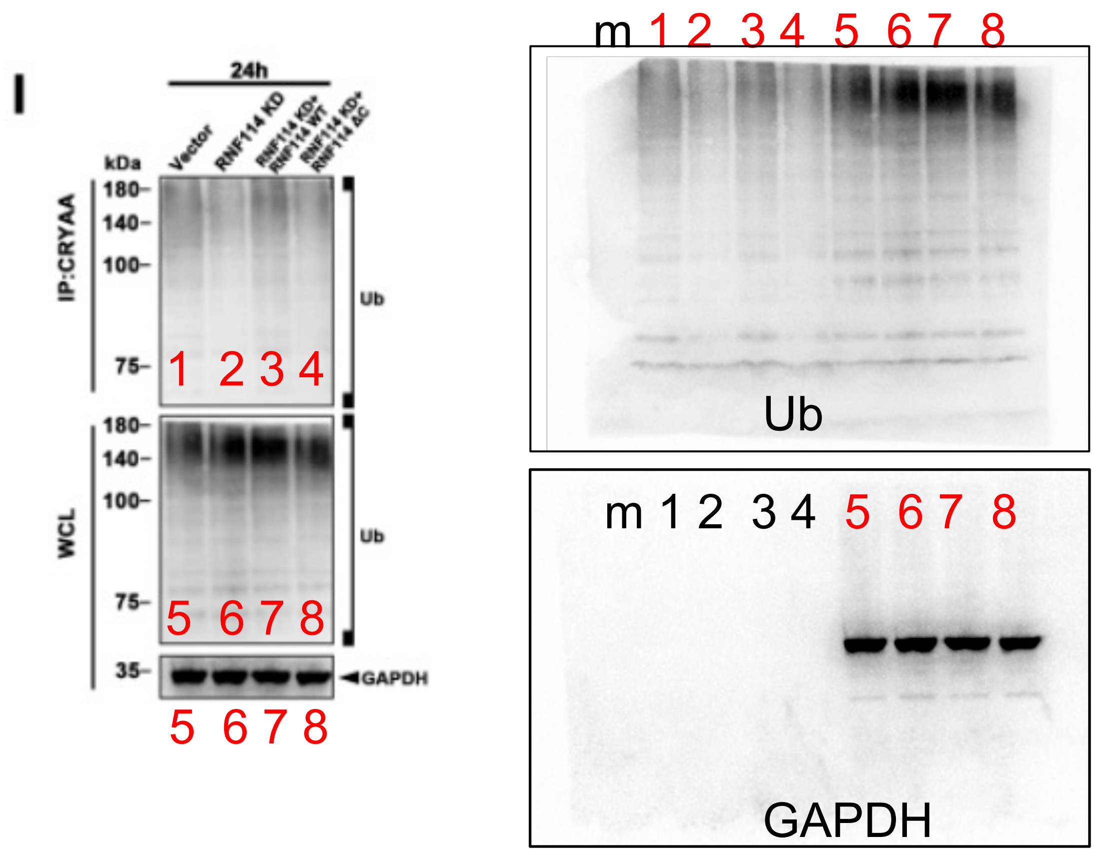

Fig. 4. J.

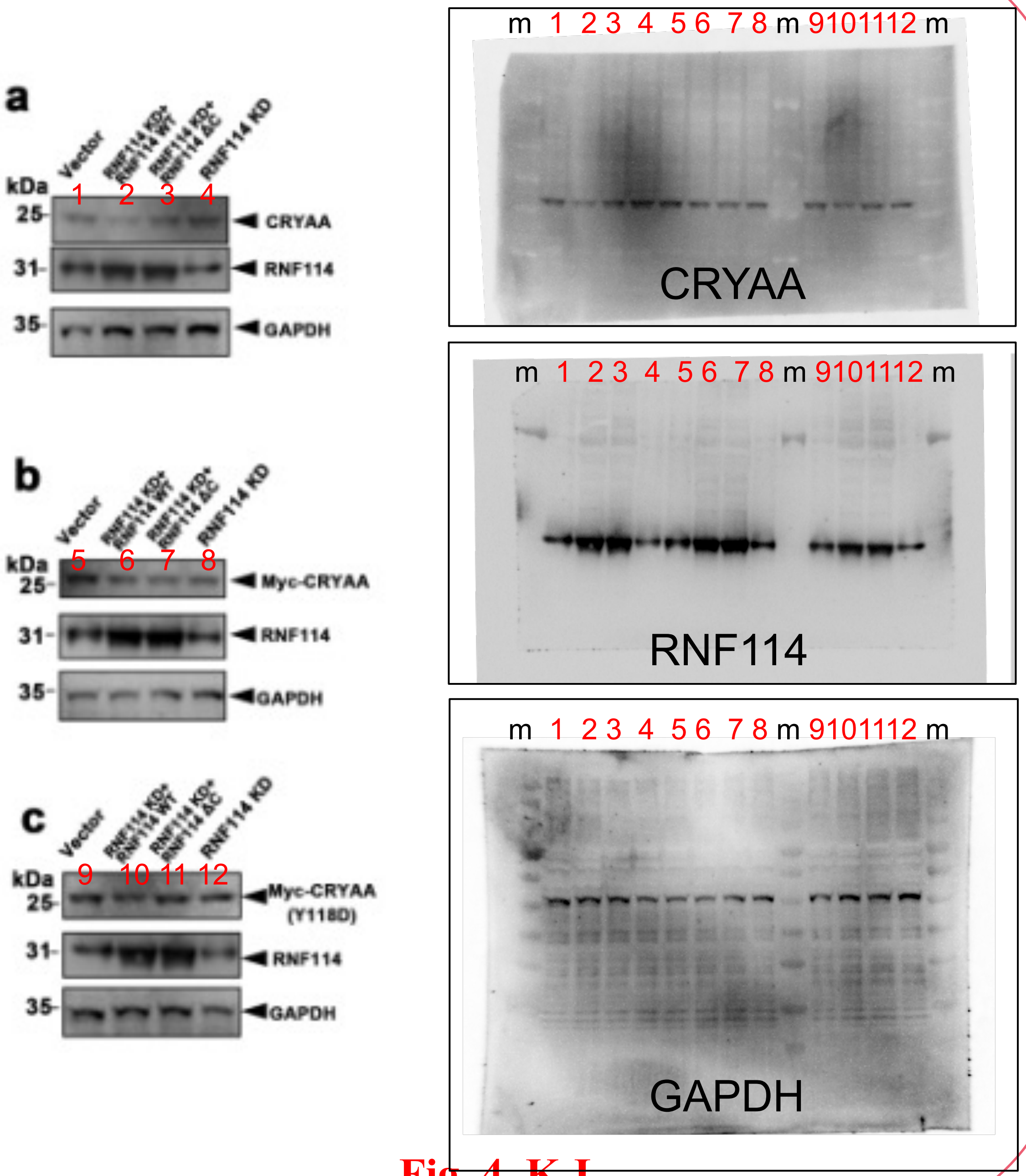

Fig. 4. K-L.

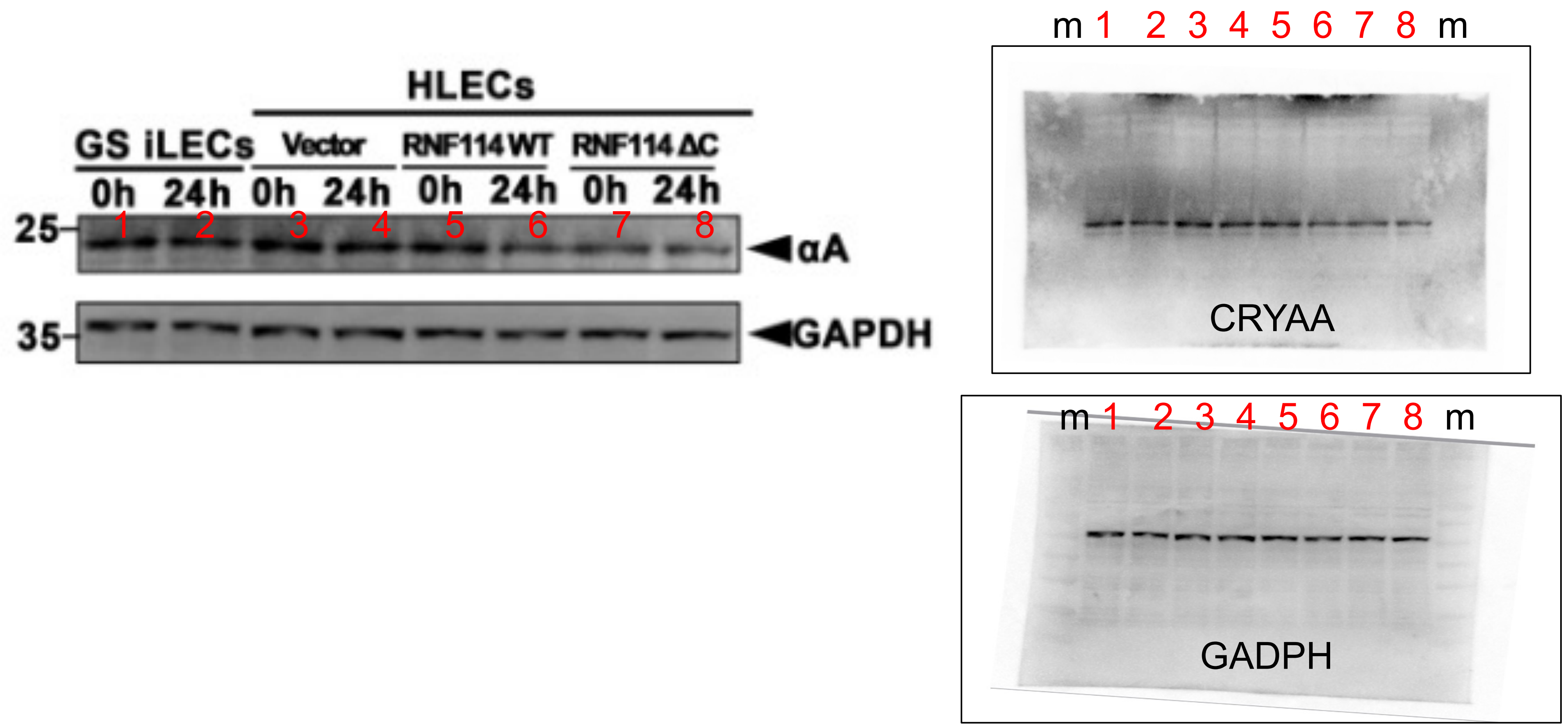
HLECs
GS iLECs
Vector
RNF114 WT
RNF114 ΔC
0h 24h 0h 24h 0h 24h 0h 24h
1 2 3 4 5 6 7 8
25
35
αA
GAPDH
m 1 2 3 4 5 6 7 8 m
CRYAA
m 1 2 3 4 5 6 7 8 m
GADPH

Fig. 5 C.

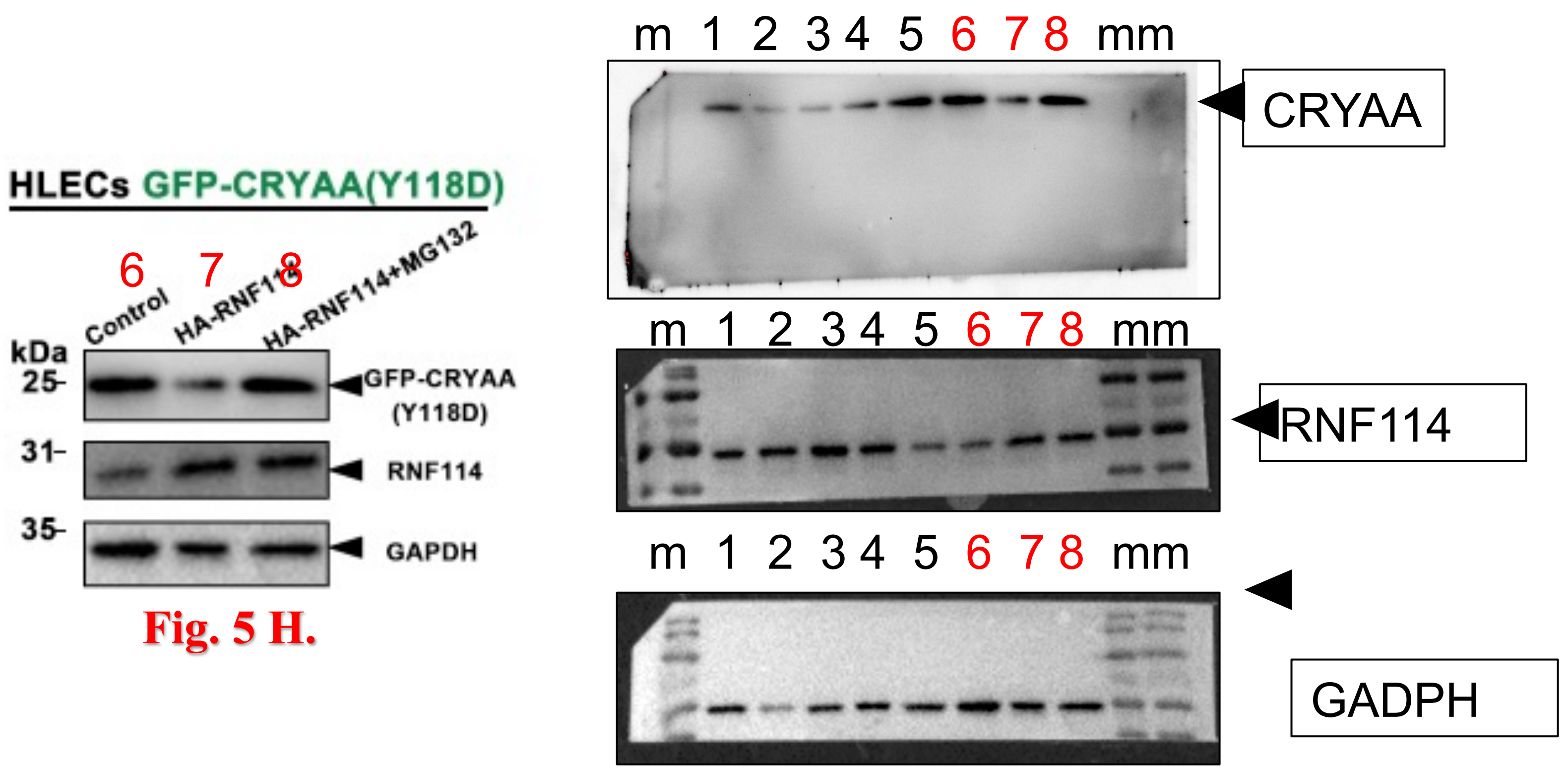
HLECs GFP-CRYAA(Y118D)
6 7 8
Control
HA-RNF114
HA-RNF114+MG132
kDa
25-
31-
35-
GFP-CRYAA (Y118D)
RNF114
GAPDH
m 1 2 3 4 5 6 7 8 mm
CRYAA
m 1 2 3 4 5 6 7 8 mm
RNF114
m 1 2 3 4 5 6 7 8 mm
GADPH

Fig. 5 H.

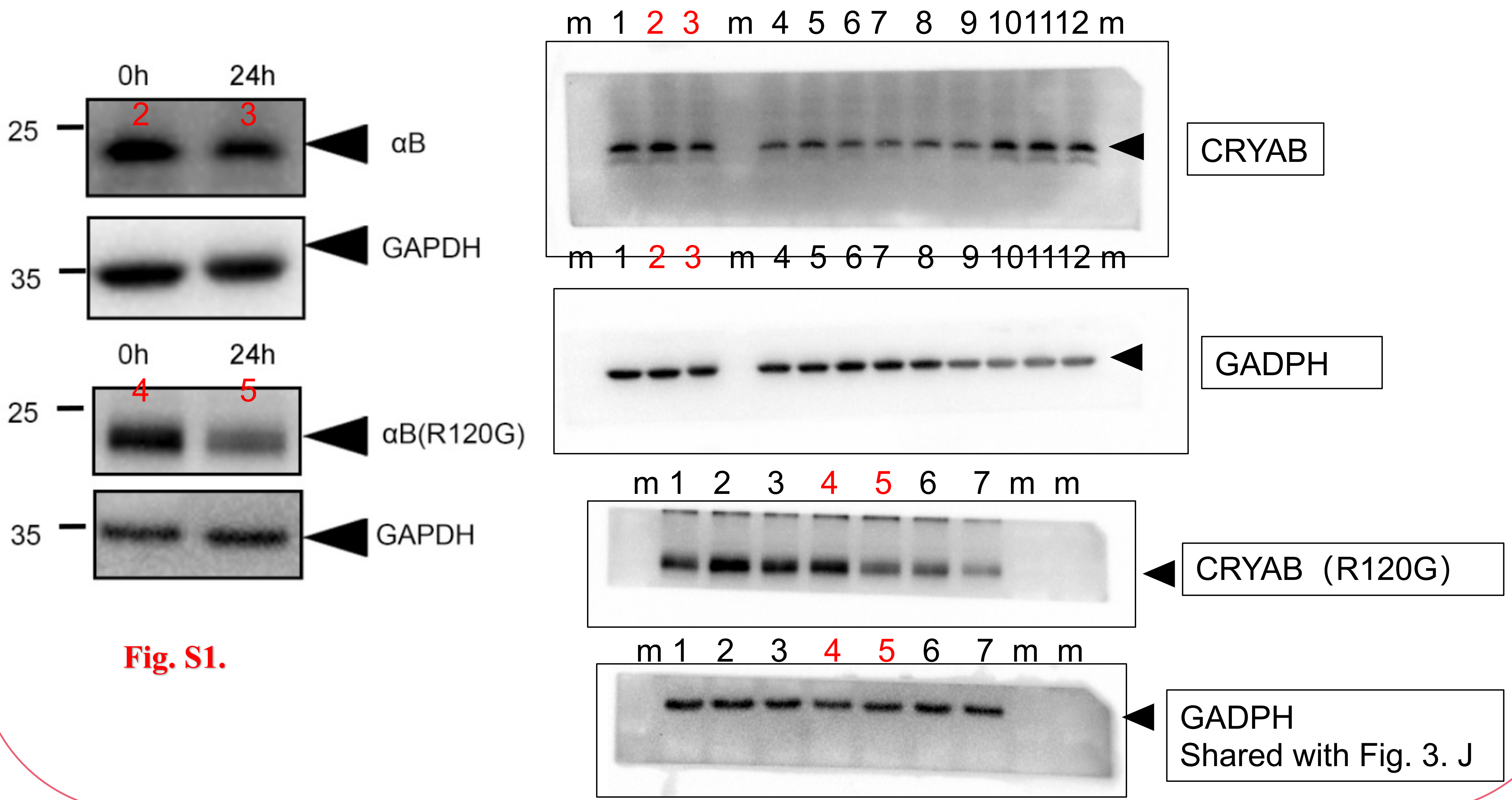
0h
24h
2
3
25
αB
GAPDH
35
0h
24h
4
5
25
αB(R120G)
35
GAPDH
m 1 2 3 m 4 5 6 7 8 9 10 11 12 m
CRYAB
m 1 2 3 m 4 5 6 7 8 9 10 11 12 m
GADPH
m 1 2 3 4 5 6 7 m m
CRYAB (R120G)
m 1 2 3 4 5 6 7 m m
GADPH
Shared with Fig. 3. J

Fig. S1.
